# Intravenous Fluid of Choice in Major Abdominal Surgery: A Systematic Review

**DOI:** 10.1155/2020/2170828

**Published:** 2020-08-03

**Authors:** Seechad Noonpradej, Osaree Akaraborworn

**Affiliations:** ^1^Department of Surgery, Faculty of Medicine, Prince of Songkla University, Hat Yai, Songkhla 90110, Thailand; ^2^Division of Trauma and Critical Care, Department of Surgery, Faculty of Medicine, Prince of Songkla University, Hat Yai, Songkhla 90110, Thailand

## Abstract

**Background:**

Intravenous fluid therapy plays a role in maintaining the hemodynamic status for tissue perfusion and electrolyte hemostasis during surgery. Recent trials in critically ill patients reported serious side effects of some types of fluids. Since the most suitable type of fluid is debatable, a consensus in perioperative patients has not been reached.

**Method:**

We performed a systematic review of randomized control trials (RCTs) that compared two or more types of fluids in major abdominal surgery. The outcomes were related to bleeding, hemodynamic status, length of hospital stay, and complications, such as kidney injury, electrolyte abnormality, major cardiac adverse event, nausea, vomiting, and mortality. A literature search was performed using Medline and EMBASE up to December 2019. The data were pooled to investigate the effect of fluid on macrocirculation and intravascular volume effect.

**Results:**

Forty-three RCTs were included. Eighteen fluids were compared: nine were crystalloids and nine were colloids. The results were categorized into macrocirculation and intravascular volume effect, microcirculation, anti-inflammatory parameters, vascular permeability, renal function (colloids), renal function and electrolytes (crystalloids), coagulation and bleeding, return of bowel function, and postoperative nausea vomiting (PONV). We found that no specific type of fluid led to mortality and every type of colloid was equivalent in volume expansion and did not cause kidney injury. However, hydroxyethyl starch and dextran may lead to increased bleeding. Normal saline can cause kidney injury which can lead to renal replacement therapy, and dextrose fluid can decrease PONV.

**Conclusion:**

In our opinion, it is safe to give a balanced crystalloid as the maintenance fluid and give a colloid, such as HES130/0.4, 4% gelatin, or human albumin, as a volume expander.

## 1. Introduction

Many factors affect the outcome of elective surgery. Beyond the nature of the primary disease and the surgical factors, intravenous fluid therapy and inotropic drugs play a role in maintaining the hemodynamic status for tissue perfusion and electrolyte hemostasis [1, 2].

The first intravenous fluid was invented about 200 years ago and evolved progressively during world wars to replace blood plasma by adding a complex sugar, protein, and colloids. [3] While believing that 0.9% sodium chloride (NaCl) is physiologic [4] and synthetic colloids are more effective than crystalloids in restoring plasma volume [5], they are widely used for resuscitation and maintenance purposes.

During recent decades, previous knowledge has been questioned. First, it was discovered that endothelial glycocalyx is the key structure to regulate microvascular hemodynamics, not oncotic pressure. These studies have led to a revised Starling principle and a new approach to vascular fluid dynamics [6]. Second, many large trials in critically ill patients and subsequent meta-analyses report potential clinical side effects of IV fluids, especially 0.9% NaCl which is associated with the development of metabolic acidosis that results in kidney injury and increases mortality rates [7, 8]. Synthetic colloids were also reported to cause side effects in kidney function and hemostasis [9–11]. Therefore, the use of all hydroxyethyl starches (HES) became restricted in critical illness, renal failure, or coagulopathy by the European Medicine Agency in 2013 and in sepsis patients by the Survival Sepsis Campaign 2012 [12]. Balanced crystalloids are currently the first choice of resuscitation in critically ill patients [2]. This knowledge has been applied to perioperative settings even though the results are inconsistent from the small number of studies with different physiological changes [1].

Our goal was to systematically review the latest evidence of perioperative intravenous fluid therapy in major abdominal surgery with a focus on the types of fluids. Volume, administration technique, and surgery beyond the abdominal field were not reviewed.

## 2. Methods

The Preferred Reporting Items for Systematic Reviews and Meta-Analyses (PRISMA) [13] guideline was used to conduct this systematic review.

### 2.1. Literature Search

We searched Medline (PubMed) and EMBASE (Ovid) databases on 16 December 2019. The keywords and Medical Subject Headings (MeSH) terms to search Medline were major abdominal surgery; any known intravenous fluid; and possible perioperative complication. The full search is included in Appendix S1. Search strategies were adapted for the other databases. The applied restrictions were randomized controlled trials (RCTs); English only; age more than 18 years; and human trial. The year of publication was not restricted.

### 2.2. Study Selection/Inclusion and Exclusion

Two levels of screening were used independently by two reviewers (SN and OA). First, the titles and abstracts of the included studies were screened and then the full text was reviewed. The included studies followed these inclusion criteria: (1) the population of patients was more than 18 years old and had undergone elective major abdominal surgery which was defined as any operation with peritoneum cavity exposure with resection and/or anastomosis; (2) intervention using two or more types or doses of intravenous fluids; and (3) the reported outcomes related to bleeding, hemodynamic status, length of hospital stay, and complications such as kidney injury, electrolyte abnormality, major cardiac adverse event, nausea, vomiting, and mortality. The excluded articles were duplicate or retracted studies, organ donor or animal studies, case reports, and case series. Any difference of opinion was resolved by discussion.

### 2.3. Data Extraction and Quality Assessment

Two authors (SN and OA) extracted data into a data sheet. The extracted data included type of surgery, number of patients, fluid regimen, and the primary and secondary outcomes of each paper. The quality of the studies was independently assessed with the Cochrane tool to assess the risk of bias for RCTs [14] in the following domains: randomization method; allocation concealment; blinding; data completeness; and publication bias. Any disagreement was resolved by discussion.

### 2.4. Data Analysis

The studies that compared the microcirculation and intravascular volume effect between colloid and crystalloid were selected for analysis. The total intraoperative volume to achieve hemodynamic parameters was used to represent the effect of crystalloid and colloid on microcirculation. The standard mean difference (SMD) was used to demonstrate the effect size of the types of fluid.

## 3. Results

### 3.1. Identification of Studies

**Figure 1:**
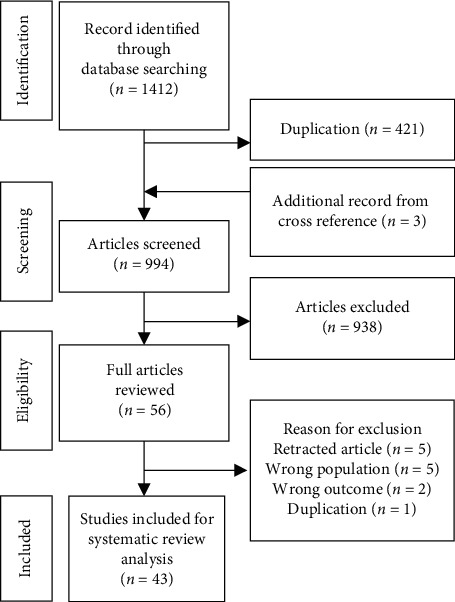
Flowchart of study search, screening, and selection.

The initial search in Medline (PubMed) and EMBASE (Ovid) identified 1,412 articles of which 421 are duplications. A further 938 were excluded because they did not fulfill the selection criteria. Fifty-six articles were selected for full-text reading. Thirteen articles were then excluded for the reasons described in Figure 1. Three additional RCT studies [15–17] were added after a cross-reference review.

### 3.2. Study Characteristics and Patient Populations

A total of 43 RCTs were included. The total numbers of patients in the included studies varied from 21 to 259 patients. Most of the studies reported around 30 patients per intervention. The types of surgery included cystectomy, radical prostatectomy, hepatectomy, laparoscopic/open colorectal surgery, gastrectomy, open abdominal aortic aneurysm repair, laparoscopic/open cholecystectomy, kidney transplantation, and liver transplantation. The main characteristics of the studies are shown in Table 1, and the types of study fluids are shown in Table 2. Full data sheet is shown in Table S1.

### 3.3. Quality of the Included Studies

**Figure 2:**
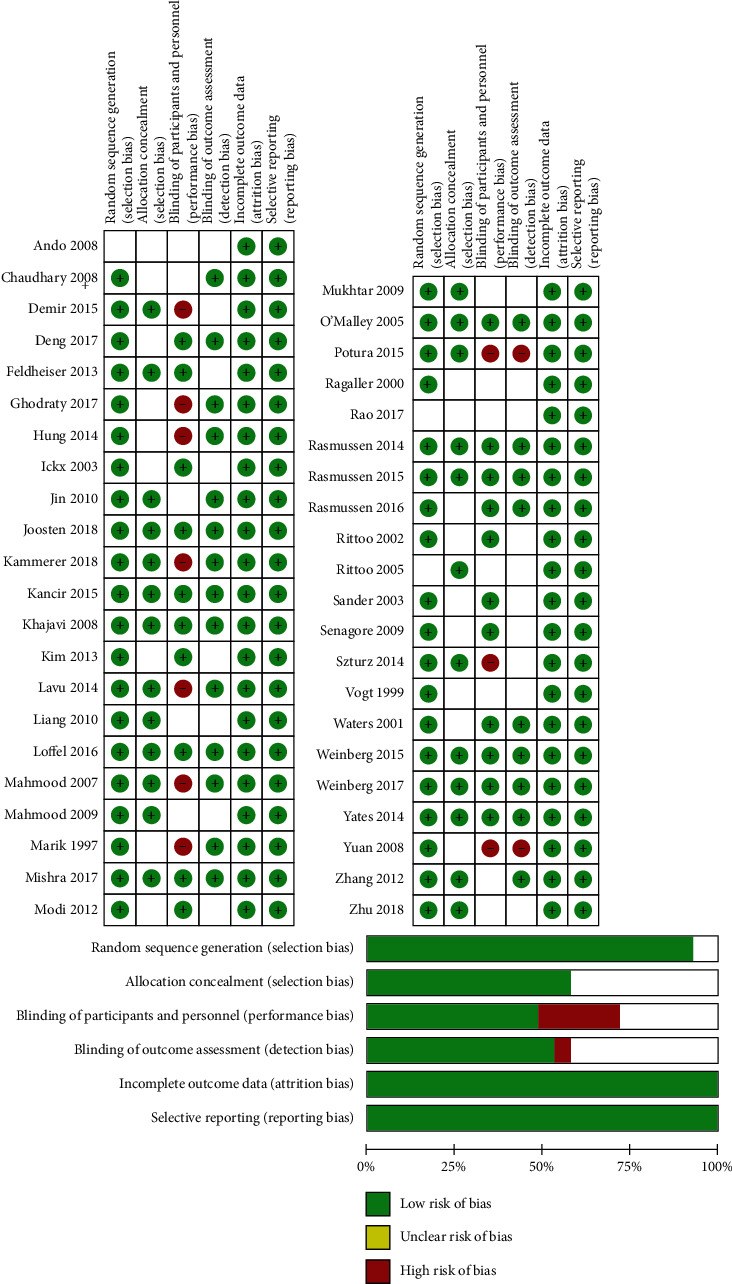
Risk of bias of original studies.

The results of the quality assessments of all studies are shown in Figure 2. Ten studies were considered high risk for blinding of participants due to safety issues. Two studies had a high risk of detection bias due to the open-label study. Most of the trials followed patients for a short period; therefore, missing data or lost to follow-up rates were low.

### 3.4. Qualitative Review

#### 3.4.1. Macrocirculation and Intravascular Volume Effect

Table 3 shows the results of 16 trials [15–17, 21, 23, 24, 27, 30, 41, 47–51, 56, 57] that reported the volume effects of fluids. Lavu et al. [30] compared 3% NaCl to lactated Ringer's solution (LRS) in patients who underwent pancreaticoduodenectomy using the fluid restriction technique and found lower perioperative intake in the 3% NaCl group (278 vs. 315 mL/kg; *p* value = 0.017) to maintain hemodynamic status. Six studies compared HES 130/0.4 (Volulyte [15–17] and Voluven [23, 27, 50]) to crystalloids. All of them reported good volume expansion according to stable hemodynamic parameters and needed both lower amounts of intraoperative fluids and inotropes to maintain hemodynamic status. Yates et al. [16] and Zhang et al. [56] who used goal-directed fluid therapy also reported colloids at crystalloid ratios of 1.6 : 1 and 1.67 : 1 to maintain the same hemodynamics in their trials. Vogt et al. [51] reported 6% HES 200/0.5 was an economical alternative to 5% human albumin for resuscitation because they had the same volume expansion effects although a lower serum colloid osmotic pressure was reported in the HES group. Two studies [24, 48] compared HES 130/0.4 to HES 200/0.5 and found no differences in the hemodynamic parameters, but HES 200/0.5 in one study [21] had a prolonged INR (1.25 ± 0.19 vs. 1.18 ± 0.09; *p* value<0.05). Ragaller et al. [41] reported HES 200 in 7.2% NaCl could restore the hemodynamics faster than HES 200 in 0.9% NaCl using pulmonary capillary wedge pressure guidance. Two studies [21, 57] compared 4% gelatin to 4.5% NaCl in 7.6% HES 40. Deng el al. [21] favored hypertonic NaCl-HES due to a more stable systemic vascular resistance index but Zhu [57] reported no significant differences in the hemodynamics.

**Figure 3:**
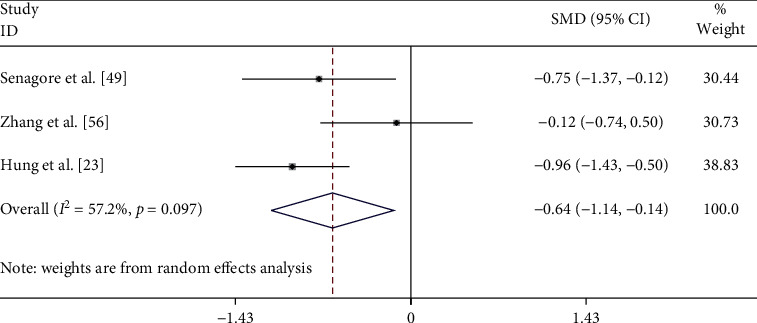
Forest plot of comparison of intraoperative fluid infusion. SMD = standard mean difference.

Among the trails in which results were related to macrocirculation and intravascular volume, eight studies [16, 17, 21, 23, 27, 49, 50, 56] compared between colloid and crystalloid. Only 3 studies mentioned the mean of intraoperative fluid volume [23, 49, 56]. The SMD was −0.638 (95% CI −1.137 to −0.138, *p*=0.012). The forest plot is shown in Figure 3.

#### 3.4.2. Microcirculation

Table 4 shows the results of five RCT studies [21, 34, 35, 46, 57] that examined the effects of fluid types on microcirculation via splanchnic circulation. Three studies were conducted in open abdominal aortic aneurysm [34, 35, 46]. Marik et al. [35] compared LRS to hetastarch (Hespan) and found that the HES group had higher gastric pH values which better represented microcirculation compared to crystalloids (*p* value<0.001). Rittoo [46] and Mahmood et al. [34] compared HES to gelatin and found that HES 200/0.62 could maintain higher gastric pH. Deng [21] and Zhu [57] compared 4% gelatin to hypertonic NaCl-HES by the acute hypervolemic infusion technique in laparoscopic colorectal surgery. Using the gastric pH value combined with the gastric-arterial CO_2_ gradient, Deng [21] reported that hypertonic NaCl-HES was better while data from Zhu [57] supported gelatin.

#### 3.4.3. Anti-Inflammatory Parameters and Vascular Permeability

Table 5 shows the results of six trials [16, 18, 33, 34, 46, 47] that studied the effects of colloids on the inflammatory process. Rittoo et al. [46, 47] and Mahmood et al. [33, 34] compared the effects of HES 200/0.62 (Elohes) and HES 130/0.4 (Voluven) to 4% gelatin (Gelofusine) in four RCTs that were performed in patients who underwent open aortic aneurysm repair. Using CRP, IL-6, and the lung injury score as biomarkers of the inflammatory process and the microalbumin/creatinine (Cr) ratio to indicate glomerular microvascular permeability, they reported that Elohes could decrease the inflammatory process by decreasing the CRP level which led to decreased microalbumin and von Willebrand factor (vWF) levels. Two studies compared medium to low molecular weight HES (HES 130/0.4 (Volulyte) [16] and HES 70/0.5 (Hespander) [18]) to balanced crystalloids. They found that both solutions did not significantly decrease the inflammation parameters and did not alter vascular permeability [18].

#### 3.4.4. Renal Function (Colloid vs. Colloid/Crystalloid)

Table 6 shows the results of 10 RCTs that reported the effects of fluids on renal function. Five trials [15–18, 27] compared colloids to crystalloids and five trials [20, 26, 33, 38, 47] compared HES to other colloids or human albumin. Ando et al. [18] compared low molecular weight HES (HES 70/0.5 or Hespander) to acetate Ringer's solution and found a significant difference in the glomerular filtration rate (GFR) and the urinary microalbumin/Cr ratio from intraoperative evaluations to discharge. Kancir et al. [27] reported no renal toxicity when HES 130/04 (Voluven) was compared to normal saline solution (NSS) using serum neutrophil gelatinase-associated lipocalin and Cr as the parameters. Three studies [15–17] compared balanced HES (Volulyte) to balanced crystalloids. The largest trial [17], which included 80 patients per group, did not show any differences in the renal function tests. In comparisons of HES to other colloids, HES 200/0.62 (Elohes) showed better renal function than 4% gelatin (Gelofusine) in two studies [33, 47] using Cr and urine albumin as the parameters. Demir et al. [20] compared HES 130/0.4 (Voluven) to 4% gelatin (Gelofusine) in patients who underwent a liver transplant and reported a nonsignificant incidence of acute kidney injury (AKI) grade I in the gelatin group (2 vs. 5). Two studies [26, 38] compared HES 130/0.4 (Voluven) to 5% human albumin and reported no differences in the renal dysfunction at neither immediate postoperation [36, 38] nor 3-month postoperation [38] using the cystatin C/Cr ratio.

#### 3.4.5. Renal Function and Electrolyte Imbalance (Balanced Solutions vs. Saline Solution)

Table 7 shows the results of eight [28, 29, 37, 39, 40, 52–54] trials that studied the effects of crystalloids on renal function. Waters et al. [52] compared the effects of NSS to LRS in patients who underwent open aortic repair. Six studies compared the effect of NSS to balanced crystalloid solutions (LRS [28, 37, 39], Plasmalyte [29, 54], and acetate buffer crystal [40]) in kidney transplant patients. The outcomes were the same as NSS which induced hyperchloremic metabolic acidosis with hyperkalemia during the intraoperative and immediate postoperative periods. One study [54] reported that hemodialysis was needed more frequently to treat hyperkalemia in the NSS group (13 vs. 4; *p* value = 0.02). Weinberg et al. [53] compared Plasmalyte to Hartmann's solution in liver resection patients. They reported no difference in renal function but Hartmann's solution showed a higher median (interquartile range (IQR)) intraoperative bleeding of 500 mL (300,638) vs. 300 mL (200,413) (*p* value = 0.03) along with coagulopathy and overall complications (56% vs. 20%; *p* value = 0.007).

#### 3.4.6. Coagulation Defect and Bleeding

Table 8 shows the results of eight studies that focused on bleeding tendency [16, 23, 25, 27, 31, 43–45]. With the exception of the Yates [16] study, most studies were small with *n* < 50. The thromboelastogram (TEG) was used as the primary outcome in all of the studies. Jin et al. [25] compared 6% HES 130/0.4 (Voluven) to 4% gelatin (Gelofusine) using LRS as the control. They found that HES delayed clot formation measured by the TEG parameters (reaction (R) time, kinetic (K) time, and *α* angle) and impaired platelet function by decreased function of coagulation factors VIII : C and vWF. Jin et al. [25] also demonstrated that gelatin reduced clot quality at one hour after loading that was indicated by a decreased TEG maximum amplitude (MA) value. Liang et al. [31] compared HES 200/0.5 to HES 130/0.4 in laparoscopic colectomy in the preload infusion technique. He found that HES 200/0.5 resulted in an impaired TEG *R* time, MA value, and decreased expressions of GPIIb/IIIa and CD62P (*p* value<0.05). Three studies [16, 23, 43] compared HES 130/0.4 to balanced crystalloids. Yates et al. [16] did not find a significant difference in the TEG parameters while two other reports [23, 43] found impaired TEG MA and K values (*p* value<0.05) in HES 130/0.4 that was associated with a greater mean (SD) blood loss (2181 (1190) vs. 1370 (603) mL; *p* value = 0.038) [43]. Kancir et al. [28] also reported greater mean (SD) bleeding when HES 130/0.4 (Voluven) was compared to NSS (1256 mL (669) vs. 747 mL (331); *p* value = 0.008). Rasmussen et al. also reported that 5% human albumin [45] and Dextran70 [44] affected TEG MA. Dextran70 was also associated with the incidence of significant bleeding (>1500 mL) in cystectomy compared to LRS (*n* (%): 11 (58) vs. 4 (22); *p* value = 0.04) without significant difference in the amounts of mean blood loss (2339 vs. 1822 mL; *p* value = 0.27) [44].

#### 3.4.7. Return to Bowel Function

Table 9 shows four studies that reported bowel function [16, 22, 32, 56]. Loffel et al. [32] compared chloride-depleted glucose solution 5% (G5K) to Ringer's maleate solution and found that G5K could enhance bowel recovery time by 38 hours. Two studies that compared 6% HES 130/0.4 to balanced crystalloid reported faster bowel recovery according to the first flatus time (86 ± 7.2 vs. 95 ± 9.1; *p* value<0.03) [56] and (73.4 ± 20.8 vs. 86.7 ± 20.8; *p* value = 0.006) [22]. In contrast, Yates et al. [16] conducted a large trial (*n* = 202) that compared balanced crystalloid to balanced 6% HES 130/0.4 (Volulyte). The results showed no difference in the number of patients who tolerated diet at postoperative day 5 (30% vs. 32%) or in the time to first flatus.

#### 3.4.8. Postoperative Nausea Vomiting (PONV)

Table 10 shows four studies that reported the effects of fluid on PONV [19, 22, 36, 42]. Chaudary et al. [19] used preoperative intravenous volume loading by LRS and hetastarch. They found that both fluids decreased the rate of PONV and vomiting at four hours after operation compared to the IV restricted group. Two studies [36, 42] that compared LRS to 5% dextrose in laparoscopic cholecystectomy showed that 5% dextrose fluid decreased the rate of PONV by more than 50%. One study [22] showed that 6% HES 130/0.4 decreased the vomiting rate compared to LRS (11% vs. 3%; *p* value = 0.266) in gastrointestinal surgery.

#### 3.4.9. Other

Yuan et al. [55] compared 20% human albumin to NSS in hypoalbuminemia patients in major abdominal surgery during postoperative days 0–2 and found no clinical or albumin level difference to postoperative day 7. Senagore et al. [49] compared 6% hetastarch in a balanced salt solution to LRS in patients who underwent laparoscopic colorectal surgery with goal-directed therapy. They reported an increased mean number of complications per patient (2 ± 1.7 vs. 4.4 ± 4) and a prolonged length of hospital stay of 6 hours in the hetastarch group. Feldheiser [15] compared 6% HES 130/04 (Volulyte) to balanced crystalloid and reported a higher mortality rate at 3 months after operation in patients who received HES (0 vs. 5; *p* value = 0.051). However, 4 of the 5 had progressive diseases, and Joosten [17] reported a higher incidence of anastomosis leakage in the crystalloid (Plasmalyte) group than in the colloid (Volulyte) group (8 vs. 0; *p* value = 0.046).

## 4. Discussion

Nowadays, the type of fluid therapy in perioperative settings is still debatable concerning the risks and benefits. The data from small single-center studies are still inconsistent. This systematic review compares each type of fluid for perioperative fluid therapy in major abdominal surgery. We found large heterogeneous outcomes due to various types of fluids compared (both colloids and crystalloids), variations in the fluid therapy protocols, types of abdominal surgery, and different parameters in outcome measurement. We attempted to group them into topics of interest.

Restoring and maintaining tissue perfusion is the primary goal of fluid therapy. In the present review using the parameters of lower fluid intake and greater hemodynamic stability, the macrocirculation or volume expansion effect showed more positive results in the colloid group compared to the crystalloid group with SMD of −0.638 (95% CI −1.137 to −0.138, *p*=0.012). A lower fluid balance can decrease the incidence of complications from volume overload such as ileus, pulmonary edema, and impaired wound healing. [58] Complications from higher colloid intake were demonstrated in the Senagore trial [49] which was the first study to demonstrate goal-directed therapy using colloids compared to crystalloids. It was reported that the hetastarch group had a significantly higher volume compared to the crystalloid results that resulted in a high frequency of total postoperative complications and longer length of stay. The authors could not identify the cause of this event. When each colloid was compared, there were no differences in hemodynamic outcomes. In our opinion, each colloid has its initial volume expansion, colloid oncotic pressure, and half-life [59]. Hypertonic saline [30] (also with HES in hypertonic saline [21, 41]) demonstrates good volume expansion compared to an isotonic saline (and HES in 0.9% NaCl). Hypertonic saline draws water out of the intracellular compartment and into the intravascular space leading to restoration of the circulating volume with smaller volumes of fluid and reduced intracranial pressure in cases associated with traumatic brain injury [60]. However, a large trial in prehospital trauma patients demonstrated a nonsignificant higher mortality rate in the hypertonic saline group [61] which may also lead to coagulopathy, increased acidosis, hypothermia, kidney injury, and immunologic disorder [62]. Yates [16] and Zhang [24] studied the colloid to crystalloid ratios of 1 : 1.6 and 1 : 1.67, respectively, in perioperative settings. These ratios were higher compared to sepsis settings (1:1–1:3) [9, 10, 63] where the previously accepted ratio was 1 : 3 [60]. This result can be explained by endothelial dysfunction and capillary leakage in the postoperative period and sepsis [64].

Since stability of the vital signs and a decrease in the lactate level reflect macrovascular status, but not microcirculation [65], acceptance of these parameters may not be enough [66]. For example, abnormal splanchnic microcirculation may present in hemorrhage, sepsis, laparoscopic procedures, and in aortic cross clamp in aortic repair. Gastric mucosal hypoperfusion increases the production of mucosal CO_2_ (PgCO_2_) and decreases gastric mucosal pH (GpHi) [67]. These two parameters were used to demonstrate microcirculation in abdominal aortic aneurysm repair [34, 35, 46] during resuscitation with HES of different molecular weights, gelatin, and crystalloids. HES 130 and HES 200 were reported to have good properties to maintain microcirculation, especially HES 200. Two studies in laparoscopic colonic surgery attempted to compare gelatin to 4.5% NaCl in 7.6% HES 40. One study supported gelatin [57] while the other reported no difference [21]. The reason they did not use the same variables to report the results was because Deng [21] claimed that gastric pH is disturbed by carbon dioxide pneumoperitoneum. Most of the included trials supported using colloids because they were better for microcirculation. These results were supported by Wu et al. [65] who compared NSS, 3% NaCl, 4% succinylated gelatin, and 6% HES 130/0.4 in the hemorrhagic shock rat model. This animal trial reported that all of these fluids stabilized the vital signs and renal blood flow, but only HES, gelatin, and 3% NaCl restored intestinal microcirculation that was demonstrated by laser speckle contrast imaging. Human albumin and dextran also reported effects in supporting microcirculation [66].

The release of inflammatory mediators during surgery, such as C-reactive protein and tumor necrosis factor, is one of the causes of impaired endothelial barrier function due to an increase of large pores in the endothelial lining and induced glycocalyx shedding [60] which results in capillary leakage and volume maldistribution [64]. In this review, we included the in vivo anti-inflammatory effects of colloids, mostly from abdominal aneurysm repair because this operation can cause high endotoxin levels and inflammation from ischemic-reperfusion injury after aortic clamping [33, 34, 46, 47]. HES 200/0.62 is the best in reducing inflammation and decreasing capillary leakage followed by HES 130/0.4, but 4% gelatin did not show this effect. In abdominal surgery [16, 18], HES 70 and HES 130 did not show significant effects in decreasing inflammation. This type of surgery may not cause as much inflammation as aortic repair. Anti-inflammatory effects of HES that were demonstrated in animal ischemic-reperfusion model [68] found that HES inhibited firm adhesion and decreased surface expression of CD11b of leukocytes. Chen et al. [69] reported that HES 130/0.4 decreased the levels of reactive oxygen species and tumor necrosis factor, while gelatin and HES 200 did not have such effects.

Most of the studies in this review compared crystalloids to colloids, and most of the colloids were HES. We found that every colloid demonstrated abnormal clot firmness and platelet function, but none of them had an abnormal coagulogram. Abnormality in the TEG tended to increase in medium molecular weight HES compared to the lower molecular weight HES [31]. Only two trials [27, 43] reported that HES 130/0.4 (Voluven) increased intraoperative hemorrhage compared to a crystalloid. However, both trials were in urological surgery which has a high chance of bleeding due to the raw surface. These results were similar to the meta-analysis by Rasmussen et al. [70] which reported on human albumin and both high and medium molecular weight HES. Higher bleeding was found in the subgroup of noncardiac surgery using HES 130 but no significant decrease was found in the amount of bleeding compared to HES 200. After a multivariate analysis, two trials [44, 45] reported that TEG MA is the only factor that could reflect the amount of intraoperative bleeding. The mechanism of impaired coagulation by colloids was reported by de Jonge and Levi [71] through dilutional effect, molecular weight dependent reduction of vWF (acquired von Willebrand disease), factor VIII, and clot firmness. Gelatin and albumin had the least effect on coagulation among the colloid solutions [60].

For a comparison of crystalloids in perioperative renal function, the information available was mainly from kidney transplantation patients who have a very high risk for renal failure. Most studies compared a balanced crystalloid to NSS and reported similar results. NSS caused hyperchloremic metabolic acidosis and hyperkalemia in the intraoperative to postoperative periods. However, we did not find a significant difference in mortality rate, AKI, graft rejection, or kidney dysfunction. However, higher early postoperative renal replacement therapy (RRT) within 48 hours was needed to treat hyperkalemia in the Weinberg et al. trial [54]. A meta-analysis by Cochrane [72], which included 1,096 participants from 18 RCTs in major perioperative settings, also reported that increased serum creatinine, hyperkalemia, negative base excess, and low serum pH occurred in the postoperative period but most subsided within postoperative day 1. No significant incidence of long-term kidney dysfunction or mortality rate was reported. This was contrary to the results of the SALTED trial [73] (study in noncritical illness) and SMART trial [8] (study in critical illness). In these trials, resuscitation used NSS which significantly increased major adverse kidney event (compound outcome) within 30 days without a significant difference in mortality rates. A large volume of NSS was related to renal vasoconstriction [60]. All of the above information was compiled into a guideline that supports using balanced crystalloids for peri-interventional volume substitution [2]. However, there were some situations where NSS was indicated, such as the presence of cerebral edema and gastric outlet obstruction [60].

Following a report of osmotic nephrosis in kidney transplant recipients after administration of HES [74], renal function after the use of colloids became a concern. However, two studies found that HES administration had better tubular and glomerular function based on the RIFLE criteria and the level of serum Cr [33, 47]. Also, another study found a lower incidence of AKI grade 1 compared to gelatin [20]. Other trials showed no significant difference in AKI using HES compared to albumin [26, 38] or HES compared to crystalloids [18, 27]. The ALBIOS trial [63] reported no difference in mortality rate or RRT when albumin was compared to colloids in sepsis patients. Many large multicenter trials reported a higher incidence of RRT [9–11] and mortality rate [10] in the HES groups compared to crystalloids in sepsis patients, but they had defects in methodology [75]. In 2013, the CRISTAL trial [76] compared crystalloids (isotonic or hypertonic saline and balanced solution) to colloids (gelatin, dextran, HES, and albumin) in patients with hypovolemic shock. They reported a lower mortality rate at 30 days and lower need of vasopressor therapy in the colloid group. No differences were found in the incidence of RRT and AKI. Furthermore, the subgroups of each type of colloid still showed a lower mortality rate. A recent meta-analysis [77] which compared colloids to crystalloids reported a higher incidence of RRT and mortality rate in the pentastarch group. In a subgroup analysis of sepsis, colloids led to a higher incidence of RRT and mortality rate, but these outcomes were not significant in cardiac and general surgery. This might be explained by the mechanism of AKI in surgery where volume loss can be improved by adequate volume replacement. However, in septic AKI, microvascular dysfunction is the key mechanism [77]. Larger endothelial pores allow colloids to leak into the tissues leading to organ dysfunction, especially in the kidney [60]. Colloids with higher molecular weights, for example, pentastarch, are more harmful due to the long metabolism time.

In two trials, a solution of 5% dextrose fluid was compared to a nondextrose fluid to determine the incidence of PONV [36, 42]. The results showed that the 5% dextrose fluid decreased the incidence of PONV. However, these two trials were performed in laparoscopic cholecystectomy and the IV fluid protocol required a postoperative loading of <1000 mL. A meta-analysis which focused on PONV using dextrose fluids also included uncomplicated surgeries (laparoscopic gynecological surgery and laparoscopic cholecystectomy). The results showed a decreased incidence of PONV and the need for antiemetics by a mechanism related to hyperglycemia [78]. Colloids can decrease PONV, vomiting, and the need for antiemetics compared to crystalloids [19, 22] by increased mucosal perfusion [78].

Many factors can affect bowel function and the type of fluid is also one of them. In our review, colloids could enhance bowel function compared to crystalloids, but may not have clinical significance (7 [16], 9 [56], and 13 [22] hours). All of the indicated trials used a goal-directed protocol and found a significantly lower need for fluid in the colloid groups. Using more crystalloids to achieve the same clinical volume effect as colloids can be detrimental. Crystalloids have a propensity to filter across the capillary membrane. A greater expansion of extravascular volume leads to intestinal mucosal edema and delayed recovery in postoperative ileus [79]. However, colloids can generate oncotic pressure to maintain fluid in intravascular component [80].

### 4.1. Limitations

The present systematic review has some limitations that should be considered when interpreting the results. First, we had too many primary outcomes which resulted in including various types of fluids, volume administration protocols, and types of surgical procedures, which may account for the high heterogeneity of our results. Second, the trials included in this systematic review were often small and single-center studies. Third, the volume of a given fluid that may affect the outcome was not included in our review. Fourth, only major abdominal surgery was our surgical type. Therefore, the results may not apply to other types of surgery. Fifth, some types of fluid (dextran and gelatin) were restricted in Europe and America which resulted in low reliability of the data obtained. Sixth, most of the participants included were ASA class I–III. Therefore, it may be incorrect to apply this information to an emergency condition or higher ASA class. Finally, there were some flaws in our search methods which caused some important trials to be missed.

The strength of this review was we had many primary outcomes which resulted in including various types of fluids, volume administration protocols, and types of surgical procedures.

## 5. Conclusion

Perioperative fluid management depends on many factors such as patient status, type of operation, type of fluid, and administration technique. The colloids had an individual volume expansion effect, maintained microcirculation, and can be used interchangeably. Every colloid affected clot firmness and clot formation time, but only dextran significantly increased bleeding. NSS resulted in perioperative hyperchloremic metabolic acidosis and hyperkalemia which may lead to RRT compared to a balanced crystalloid. No specific type of fluid increased the mortality rate.

## Figures and Tables

**Table 1:** Main characteristics of the studies included in this review.

Author, years	*N*	Age, range (average)	Sex (M/F)	ASA (N)	Fluid A	Fluid B	Fluid C	Operation	Primary outcome/primary end point
Ando et al. [18], 2008	21	67 (60, 70)	12/9	I/II (9/12)	Acetate Ringer	HES70/0.5 (Hespander)	—	Major abdominal surgery	Urinary microalbumin/creatinine ratio
Chaudhary et al. [19], 2008	60	41 ± 11.06	—	I/II	LRS 2 mL/kg	LRS 12 mL/kg	4.5% hetastarch 12 mL/kg	Open cholecystectomy	PONV at 24 hours (VAS)
Demir et al. [20], 2018	36	42.72 ± 13.25	25/11	II/III/IV (21/8/7)	6% HES130/0.4 (Voluven)	4% gelatin (Gelofusine)	—	Living donor liver transplant	Renal function (Cr, BUN, and GFR)
Deng et al. [21], 2017	36	40–80	20/16	I/II	LRS	4% gelatin (Gelofusine)	4.5% NaCl in 7.6% HES40	Laparoscopic colonic surgery	Mucosal blood flow (Pg-aCO2)
Feldheiser et al. [15], 2013	48	52.5 (45.5, 59)	—	I/II/III (4/24/20)	Jonosteril	6% HES130/0.4 (Volulyte)	—	Cytoreductive surgery	Amount of fluid
Ghodraty et al. [22], 2017	91	53.2 ± 12.3	60/31	II/III (38/53)	LRS	6% HES130/0.4 (Voluven)	—	GI surgery	Presence of bowel function
Hung et al. [23], 2014	80	48 ± 10.7	48/32	—	LRS	6% HES130/0.4 (Voluven)	—	Major abdominal surgery	Thromboelastogram
Ickx et al. [24], 2003	40	62 (47–72)	39/1	II/III	6% HES130/0.4 (Voluven)	6% HES200/0.5 (HAES-steril)	—	Major abdominal surgery	Plasma substitution effect (CO, RVEDV)
Jin et al. [25], 2010	42	49 ± 10	15/27	I/II	LRS	6% HES130/0.4 (Voluven)	4% gelatin (Gelofusine)	Gastrectomy	Thromboelastogram
Joosten et al. [17], 2018	160	62 (48–70)	96/84	II/III (93/67)	Plasmalyte	6% HES130/0.4 (Volulyte)	—	Major abdominal surgery	Postoperative complication at day 2
Kammerer et al. [26], 2018	100	70 (61–75)	81/19	I/II/III/IV (6/38/63/2)	5% human albumin	6% HES130/0.4 (Voluven)	—	Cystectomy	Serum cystatin C ratio (preoperative/ postoperative day 90)
Kancir et al. [27], 2015	36	64 (4.8)	—	—	NSS	6% HES130/0.4 (Voluven)	—	Radical cystectomy	Urine NGAL
Khajavi et al. [28], 2008	54	40 ± 14	—	—	NSS	LRS	—	Living donor kidney transplant	Serum potassium and pH
Kim et al. [29], 2013	60	46 ± 12	38/22	III/IV	NSS	Plasmalyte	—	Living donor kidney transplant	Renal function
Lavu et al. [30], 2014	259	68.3 (25–91)	39%/46%	III (167)	LRS	3% sodium chloride	—	Pancreaticoduodenectomy	Postoperative complication
Liang et al. [31], 2010	35	57 ± 8	15/20	I/II	6% HES130/0.4 (Voluven)	6% HES200/0.5 (HAES-steril)	—	Laparoscopy-assisted radical colectomy	Thromboelastogram
Loffel et al. [32], 2016	44	71.5 (33–82)	30/14	II/III (28/16)	Ringer maleate	Chloride-depleted glucose solution 5% (G5K)	—	Cystectomy	First defecation
Mahmood et al. [33], 2007	62	72 (7)	50/12	—	6% HES 200/0.62 (Elohes)	6% HES130/0.4 (Voluven)	4% gelatin (Gelofusine)	Open AAA repair	Splanchnic perfusion (gastric pH)
Mahmood et al. [34], 2009	62	72 (7)	50/12	—	6% HES 200/0.62 (Elohes)	6% HES130/0.4 (Voluven)	4% gelatin (Gelofusine)	Open AAA repair	Renal function (Cr, GFR)
Marik et al. [35], 1997	30	—	—	—	LRS	HES670/0.75 (hetastarch)	—	Open AAA repair	Maximal change of gastric pH
Mishra et al. [36], 2017	100	39.6 ± 11.54	28/72	I/II (81/19)	NSS	5% Dextrose	—	Laparoscopic cholecystectomy	Incidence of PONV
Modi et al. [37], 2012	72	18–62	-	—	NSS	LRS	—	Living donor kidney transplant	Acidosis, potassium
Mukhtar et al. [38], 2009	40	51 ± 6	35/5	—	5% human albumin	6% HES130/0.4 (Voluven)	—	Living donor liver transplant	Creatinine clearance at 24 hours
O'Malley et al. [39], 2005	51	44 ± 13	32/19	—	NSS	LRS	—	Kidney transplant	Cr at postoperative day 3
Potura et al. [40], 2015	148	56 ± 13	95/53	—	NSS	Elomel-Isoton	—	Cadaveric kidney transplant	Perioperative hyperkalemia
Ragaller et al. [41], 2000	29	68.4 ± 8.5	26/3	I/II/III	6% HES200/0.5 + 0.9%NaCl	6% HES200/0.5 + 7.2%NaCl	—	Open AAA repair	Amount of fluid to restore PCWP
Rao et al. [42], 2017	112	19–60	—	I/II	LRS	5% dextrose	—	Laparoscopic cholecystectomy	Incidence of PONV
Rasmussen et al. [43], 2014	33	64.1 (7.9)	26/7	I/II/III	LRS	Dextran 70	—	Cystectomy	Thromboelastogram
Rasmussen et al. [44], 2015	37	68 (61.9–74.3)	27/10	I/II/III	LRS	5% human albumin	—	Cystectomy	Thromboelastogram
Rasmussen et al. [45], 2016	39	69 (66–72)	25/14	I/II/III	LRS	6% HES130/0.4 (Voluven)	—	Cystectomy	Thromboelastogram
Rittoo et al. [46], 2002	22	70.6 ± 2.18	15/7	—	4% gelatin (Gelofusine)	6% HES200/0.62 (Elohes)	—	Open AAA repair	Splanchnic perfusion (gastric pH)
Rittoo et al. [47], 2005	40	71.2 (6.7)	30/10	—	4% gelatin (Gelofusine)	6% HES200/0.62 (Elohes)	—	Open AAA repair	Inflammatory marker
Sander et al. [48], 2003	56	45 ± 15	—	I/II/III (16/36/4)	6% HES130/0.4 (Voluven)	6% HES200/0.5	—	Major gynecological surgery	Hemodynamic maintenance
Senagore et al. [49], 2009	64	—	—	I/II/III	Standard-LR	Goal-directed LR	Goal-directed hetastarch	Laparoscopic colonic surgery	Length of hospital stay
Szturz et al. [50], 2014	115	61 (27–87)	83/32	I/II/III/IV	LRS	6% HES130/0.4 (Voluven)	—	Major urological surgery	Efficiency of volume expansion
Vogt et al. [51], 1999	48	65 (7.1)	—	I/II/III (4/33/13)	5% human albumin	6% HES200/0.5	—	Major urological surgery	Hemodynamic stability effect
Waters et al. [52], 2001	66	69.8 ± 8.7	—	I-IV (III)	NSS	LRS	—	Open AAA repair	Change in base excess
Weinberg et al. [53], 2015	60	63 (38–85)	36/24	I/II/III (1/26/33)	Hartmann solution	Plasmalyte	—	Major liver resection	Immediate postoperative base excess
Weinberg et al. [54], 2017	49	49 (26–67)	33/16	—	NSS	Plasmalyte	—	Cadaveric kidney transplant	Postoperative hyperkalemia 48 hours
Yates et al. [16], 2014	202	72 (56–88)	117/85	I/II/III/IV (20/119/62/1)	Hartmann solution	6% HES130/0.4 (Volulyte)	—	Colorectal surgery	GI morbidity at postoperative day 5
Yuan et al. [55], 2008	127	56.1 ± 15.3	69/58	—	NSS	20% human albumin	—	Major abdominal surgery	Albumin level
Zhang et al. [56], 2012	60	56.7 ± 6.9	42/18	I/II (32/28)	Restricted-LR	Goal-directed LR	Goal-directed HES130/0.4	GI surgery	Length of hospital stay
Zhu et al. [57], 2018	71	73 ± 7	46/34	I/II	LRS	4% gelatin (Gelofusine)	4.5% NaCl in 7.6% HES40	Laparoscopic colonic surgery	Splanchnic perfusion (gastric pH)

HES = hydroxyethyl starch, LRS = lactated Ringer's solution, VAS = visual analog scale, BUN = blood urea nitrogen, Cr = creatinine clearance, GFR = glomerular filtration rate, CO = cardiac output, RVEDV = right ventricular end diastolic volume, NSS = normal saline solution, AAA = abdominal aortic aneurysm, PCWP = pulmonary capillary wedge pressure, and PONV = postoperative nausea vomiting.

**Table 2:** Characteristics of perioperative fluids included in this review.

Fluid	Na^+^	K^+^	Cl^−^	Ca^2+^	Mg^2+^	HCO3^−^	Buffer	Glucose (g/L)	Other	Molecular wt (kDa)/C2 : C6 ratio	Osmolarity	Oncotic pressure (mmHg)	pH (in vitro)	Initial volume expansion (%)	Persistence in the body (days)	Maximal daily dose (per kg)
Plasma	140	5	100	4.4	2	4.4	Lactate 1	—	—	—	285	—	7.4	—	—	—
0.9% NaCl (NSS)	154	—	154	—	—	—	—	—	—	—	308	—	6	—	—	—
3% NaCl	513	—	513	—	—	—	—	—	—	—	1026	—	4.5	—	—	—
5% Dextrose	—	—	—	—	—	—	—	50	—	—	252	—	4.5	—	—	—
5% Dextrose/ 0.45%NaCl	77	—	77	—	—	—	—	50	—	—	406	—	4	—	—	—
Lactated Ringer solution (Hartmann's solution, LRS)	130	4	109	3	—	—	Lactate 28	—	—	—	273	—	6.5	—	—	—
Plasmalyte	140	5	98	—	3	—	Acetate 27 Gluconate 23				294		7.4			
Jonosteril	137	4	110	1.6	1.2	—	Acetate 36.8	—	—	—	na	—	na	-	-	-
Ringer maleate (Ringerfundin)	145	4	127	2.5	1	—	Maleate 5 Acetate 24				na		na			
G5K solution	50	30	65	0	2	HPO4 8	Lactate 18	50	—	—	454	—	na	—	—	—
4%–5% Albumin	130–160	—	130–160	—	—	—	—	—	—	—	309	20–29	7.1	80	-	-
Dextran70	154	—	154	—	—	—	—	—	—	—	na	56–68	na	120	28–42	1.5
6% HES 670/0.75 (hetastarch)	154	—	154	—	—	—	—	—	—	670/4.5 : 1	309	25–30	5.5	100	4–6	20
6% HES 200/0.62 (Elohes)	154	—	154	—	—	—	—	—	—	200/9 : 1	na	25–30	na	110	6–7	20
6% HES 200/0.5 (Hesteril)	154	—	154	—	—	—	—	—	—	200/5 : 1	Na	30–37	na	100	3–4	33
6% HES 130/0.4 NSS (Voluven)	154	—	154	—	—	—	—	—	—	130/9 : 1	308	36	na	100	2–3	50
6% HES 130/0.4 balanced solution (Volulyte)	137	4	110	—	3	—	Acetate 34	—	—	130/9 : 1	287	na	na	100	2–3	50
6% HES 70/0.5 in balanced solution (Hespander)	105	4	92.3	2.7	—	—	Lactate 20	—	—	70/3 : 1	—	—	na	100	1–2	20
4% succinylated gelatin (Gelofusine)	154	—	125	—	—	—	—	—		30	274	—	7.1–7.7	80	2–7	—

*Note.* Electrolyte concentrations, osmolarity, and pH may be subject to small differences in other reports. HES = hydroxyethyl starch; kDa = kilodalton.

**Table 3:** Overview of randomized control trials in which the results were related to macrocirculation and intravascular volume effect categorized by primary outcome.

Author, year	Fluid compared	*N*	Operation	Conclusion
*Primary outcome: hemodynamic parameters (dynamic parameter, static parameter, and colloid oncotic pressure)*				
Vogt [51], 1999	6% HES 200/0.5 5% human albumin	48	Major urological surgery	No significant difference in static hemodynamic parameters HES has lower colloid oncotic pressure
Ragaller [41], 2000	6% HES 200/0.5 + 7.2%NaCl 6% HES 200/0.5 + 0.9%NaCl	29	Abdominal aortic aneurysm repair	Hypertonic NaCl-HES needed lower volume and restored PCWP faster than HES in NSS after aortic clamp off
Ickx [24], 2003	6% HES130/0.4 (Voluven) 6% HES200/0.5	40	Major abdominal surgery	No significant difference in dynamic hemodynamic parameter No significant difference in colloid oncotic pressure
Sander [48], 2003	6% HES130/0.4 (Voluven) 6% HES200/0.5	56	Major gynecological	No significant difference in static hemodynamic parameter No significant difference in volume needed to maintain hemodynamics
Feldheiser [15], 2013	Jonosteril 6% HES130/0.4 (Volulyte)	48	Cytoreductive surgery	HES reduced need for FFP and IV fluid to maintain hemodynamics. No significant difference in need for inotrope.
Szturz [50], 2014	LRS HES 130/0.4 (Voluven)	115	Major urological surgery	HES reduced need for FFP and IV fluid to maintain hemodynamics

*Primary outcome was another objective but also had these outcomes*				
Rittoo [47], 2005	HES 200/0.62 (Elohes) 4% gelatin (Gelofusine)	40	Abdominal aortic aneurysm repair	Lower HES intake to maintain hemodynamics compared to gelatin
Senagore [49], 2009	Standard-LRS GD-LRS GD-hetastarch balance	64	Laparoscopic colonic surgery	Lower HES intake to achieved target stroke volume
Zhang [56], 2012	Restricted-LRS GD-LRS GD-HES 130/0.4 Crystalloid : colloid ratio = 1.67 : 1	60	GI surgery	HES reduced need of IV fluid to maintain hemodynamics
Hung [23], 2014	LRS 6% HES 130/0.4 (Voluven)	80	Major abdominal surgery	HES reduced need of IV fluid to maintain hemodynamics
Lavu [30], 2014	LRS 3% NaCl	259	Pancreaticoduodenectomy	3% NaCl reduced need of IV fluid to static maintain hemodynamics
Yates [16], 2014	Hartmann's solution 6% HES 130/0.4 (Volulyte) Crystalloid : colloid ratio = 1.6 : 1	202	Colorectal surgery	HES reduced IV fluid to maintain hemodynamics
Kancir [27], 2015	NSS 6% HES 130/0.4 (Voluven)	36	Radical prostatectomy	No significant difference in fluid need to maintain hemodynamics
Deng [21], 2017	LRS 4% gelatin (Gelofusine) 4.5% NaCl in 7.6% HES 40	36	Laparoscopic colonic surgery	HS-HES can prolong effect of volume expansion and decreased systemic vascular resistance index
Joosten [17], 2018	Plasmalyte 6% HES130/0.4 (Volulyte)	160	Major abdominal surgery	HES reduced need of IV fluid to maintain hemodynamics by dynamic monitoring

HES = hydroxyethyl starch, NaCl = sodium chloride, PCWP = pulmonary capillary wedge pressure, NSS = normal saline solution, LRS = lactated Ringer's solution, FFP = fresh frozen plasma, GD = goal-directed therapy, and GI = gastrointestinal.

**Table 4:** Overview of randomized control trials related to microcirculation categorized by primary outcome.

Author, year	Fluid compared	*N*	Operation	Conclusion
*Primary outcome: Gastric* pH *and Pg-aCO2*				
Marik [35], 1997	LRS HES 670/0.75 (hetastarch)	30	Abdominal aortic aneurysm repair	HES 670/0.75 improved splanchnic mucosal blood flow (gastric pH)
Rittoo [46], 2002	6% HES 200/0.62 (Elohes) 4% gelatin (Gelofusine)	22	Abdominal aortic aneurysm repair	HES 200/0.5 improved splanchnic mucosal blood flow (gastric pH)
Mahmood [34], 2009	6% HES 200/0.62 (Elohes) 6% HES 130/0.4 (Voluven) 4% gelatin (Gelofusine)	62	Abdominal aortic aneurysm repair	HES is better than gelatin but HES 200/0.62 was the best in decreasing gastric pH after clamp off
Deng [21], 2017	LRS 4% gelatin (Gelofusine) 4.5% NaCl in 7.6% HES40	36	Laparoscopic colonic surgery	No significant splanchnic mucosal blood flow (Pg-aCO_2_)
Zhu [57], 2018	LRS 4% gelatin (Gelofusine) 4.5% NaCl in 7.6% HES 40	71	Laparoscopic colonic surgery	4% gelatin was the best in maintaining gastric pH > 7.32 for more than 60 minutes of operation

HES = hydroxyethyl starch, NaCl = sodium chloride, and LRS = lactated Ringer's solution.

**Table 5:** Overview of randomized control trials related to anti-inflammatory parameters and vascular permeability categorized by primary outcome.

Author, year	Fluid compared	*N*	Operation	Conclusion
*Primary outcome: inflammatory mediators (IL-6, CRP, ICAM-1, and vWF) and vascular permeability (urine albumin/Cr ratio)*				
Rittoo [46], 2002	6% HES 200/0.62 (Elohes) 4% gelatin (Gelofusine)	22	Abdominal aortic aneurysm repair	HES 200/0.62 lowered CRP but no difference in IL-6 level
Rittoo [47], 2005	6% HES 200/0.62 (Elohes) 4% gelatin (Gelofusine)	40	Abdominal aortic aneurysm repair	HES 200/0.62 decreased inflammatory process and reduced endothelial activation
Mahmood [33], 2007	6% HES 200/0.62 (Elohes) 6% HES 130/0.4 (Voluven) 4% gelatin (Gelofusine)	62	Abdominal aortic aneurysm repair	Less derangement in marker of glomerular and tubular function in HES 200/0.62 and HES130/0.4
Ando [18], 2008	HES 70/0.5 (Hespander) Acetate Ringer	20	Major abdominal surgery	No significant difference in inflammatory markers and vascular permeability

*Primary outcome was another objective but also had these outcomes*				
Mahmood [34], 2009	6% HES 200/0.62 (Elohes) 6% HES 130/0.4 (Voluven) 4% gelatin (Gelofusine) Endotoxin level increased in gelatin group	62	Abdominal aortic aneurysm repair	HES 200/0.62 mostly decreased inflammatory process (CRP, but not lung injury score)
Yates [16], 2014	6% HES130/0.4 (Volulyte) Hartmann's solution	202	Colorectal surgery	No significant difference in inflammatory marker

IL-6 = interleukin-6; CRP = C-reactive protein; ICAM-1 = intercellular adhesion molecule-1; vWF = von Willebrand factor; Cr = creatinine; HES = hydroxyethyl starch; GI = gastrointestinal; POD = postoperative day.

**Table 6:** Overview of randomized control trials related to renal function (colloid vs. colloid/crystalloid) categorized by primary outcome.

Author, year	Fluid compared	*N*	Operation	Conclusion
*Primary outcome: renal function (serum Cr, GFR, neutrophil gelatinase-associated lipocalin, urine albumin, and cystatin C)*				
Mahmood [33], 2007	6% HES 200/0.62 (Elohes) 6% HES 130/0.4 (Voluven) 4% gelatin (Gelofusine)	62	Abdominal aortic aneurysm repair	Less derangement in marker of glomerular filtration and tubular function in both HES groups No difference in AKI or RRT
Mukhtar [38], 2009	5% human albumin 6% HES 130/0.4 (Voluven)	40	Living donor liver transplant	No difference in serum Cr, CrCl, or cystatin C level No difference in AKI or RRT
Demir [20], 2015	6% HES 130/0.4 (Voluven) 4% gelatin (Gelofusine)	36	Living donor liver transplant	Significantly decreased GFR in gelatin group No difference in AKI or RRT
Kancir [27], 2015	6% HES 130/0.4 (Voluven) NSS	36	Prostatectomy	No significant difference in renal impairment by U-NGAL, P-NGAL, and serum Cr
Kammerer [26], 2018	5% human albumin 6% HES 130/0.4 (Voluven)	100	Cystectomy	No significant difference in renal impairment by cystatin C ratio, P-NGAL, and GFR

*Primary outcome was another objective but also had these outcomes*				
Rittoo [47], 2005	6% HES 200/0.62 (Elohes) 4% gelatin (Gelofusine)	40	Abdominal aortic aneurysm repair	Less derangement in marker of glomerular function in HES group
Ando [18], 2008	Acetate Ringer HES 70/0.5 (Hespander)	20	Major abdominal surgery	No difference in glomerular function (urine Albumin/ Cr ratio), GFR
Feldheiser [15], 2013	Jonosteril 6% HES130/0.4 (Volulyte)	48	Cytoreductive surgery	No significant renal function impairment (P-NGAL and Cr)
Yates [16], 2014	Hartmann's solution 6% HES130/0.4 (Volulyte)	202	Colorectal surgery	No significant renal function impairment
Joosten [17], 2018	Plasmalyte 6% HES130/0.4 (Volulyte)	160	Major abdominal surgery	No significant renal function impairment or RRT

HES = hydroxyethyl starch, AKI = acute kidney injury, RRT = renal replacement therapy, CrCl = creatinine clearance, GFR = glomerular filtration rate, NSS = normal saline solution, U-NGAL = urine neutrophil gelatinase-associated lipocalin, P-NGAL = plasma neutrophil gelatinase-associated lipocalin, and Cr = creatinine.

**Table 7:** Overview of randomized control trials related to renal function and electrolyte imbalance (balanced vs. saline solution/other balanced solutions) categorized by primary outcome.

Author, year	Fluid compared	*N*	Operation	Conclusion
*Primary outcome: renal function or electrolyte abnormality*				
Waters [52], 2001	NSS LRS	66	Abdominal aortic aneurysm repair	NSS had more hyperchloremic metabolic acidosis No difference in Cr, AKI but no K report
O'Malley [39], 2005	NSS LRS	51	Living donor kidney transplant	NSS had more hyperchloremic metabolic acidosis No difference in Cr, AKI, K, and incidence of dialysis to 6 months
Khajavi [28], 2008	NSS LRS	54	Living donor kidney transplant	NSS had more hyperchloremic metabolic acidosis NSS had higher K level postoperation; no difference in Cr level
Modi [37], 2012	NSS LRS	72	Living donor kidney transplant	NSS had more hyperchloremic metabolic acidosis NSS had higher K level postoperation; no difference in Cr level
Kim [29], 2013	NSS Plasmalyte	60	Living donor kidney transplant	NSS had more negative base excess and chloride No difference in urine output, Cr, Cl
Potura [40], 2015	NSS Acetate-buffered crystalloid (Elomel-Isoton)	148	Cadaveric kidney transplant	NSS had more negative base excess No difference in urine output, Cr, Cl, and dialysis No difference in number of patients having K level >5.4
Weinberg [53], 2015	Hartmann solution Plasmalyte	60	Major liver resection	Higher magnesium but lower calcium in Plasmalyte group No difference in base excess and Cr
Weinberg [54], 2017	NSS Plasmalyte	49	Cadaveric kidney transplant	NSS had more hyperchloremic metabolic acidosis and hyperkalemia which led to dialysis or medication treatment

NSS = normal saline solution, LRS = lactated Ringer's solution, Cr = creatinine, AKI = acute kidney injury, and K = potassium.

**Table 8:** Overview of randomized control trials related to coagulation defect and bleeding categorized by primary outcome.

Author, years	Fluid comparisons	*N*	Operation	Conclusion
*Primary outcome: coagulation*				
Jin [25], 2010	LRS 6% HES 130/0.4 (Voluven) 4% gelatin (Gelofusine)	36	Gastrectomy	HES impaired clot initiation and impaired platelet function Gelatin reduced clot firmness No difference in blood loss
Liang [31], 2010	6% HES 200/0.5 (HAES-steril6%) 6% HES 130/0.4 (Voluven)	35	Laparoscopy-assisted radical colectomy	HES 200/0.5 impaired clotting time, clot firmness, and impaired platelet function more than HES 130/0.4 No difference in blood loss
Hung [23], 2014	LRS 6% HES 130/0.4 (Voluven)	80	Major abdominal surgery	HES 130/0.4 impaired clot initiation and strength No difference in blood loss
Rasmussen [43], 2014	LRS 6% HES 130/0.4 (Voluven)	33	Cystectomy	HES 130/0.4 impaired clot strength and firmness HES 130/0.4 caused more blood loss than LRS
Rasmussen [44], 2015	Dextran70 LRS	37	Cystectomy	Dextran70 impaired clot firmness and incidence of blood loss >1500 mL No difference in mean blood loss
Rasmussen [45], 2016	5% human albumin LRS	39	Cystectomy	5% human albumin impaired clot firmness No difference in blood loss

*Primary outcome was another objective but also had these outcomes*				
Yates [16], 2014	Hartmann's solution 6% HES 130/0.4 (Volulyte)	202	Colorectal surgery	No significant difference in TEG or blood loss
Kancir [27], 2015	NSS 6% HES 130/0.4 (Voluven)	36	Radical prostatectomy	Significant blood loss in HES

HES = hydroxyethyl starch, NSS = normal saline solution, LRS = lactated Ringer's solution, and TEG = thromboelastogram.

**Table 9:** Overview of randomized control trials related to return of bowel function by primary outcome.

Author, year	Fluid compared	*N*	Operation	Conclusion
*Primary outcome: return to bowel function, ileus, passing flatus, defecation, or enteral food tolerance*				
Yates [16], 2014	Hartmann's solution 6% HES 130/0.4 (Volulyte)	202	Colorectal surgery	No difference in bowel recovery time
Loffel [32], 2016	Chloride-depleted glucose solution 5% (G5K) Ringer maleate	44	Cystectomy	G5K group could pass normal stool faster than RM (38 hours)
Ghodraty [22], 2017	LRS 6% HES 130/0.4 (Voluven)	91	GI surgery	HES 130/0.4 reduced time of postoperative ileus (13 hours)

*Primary outcome was another objective but also had these outcomes*				
Zhang [56], 2012	Restricted-LRS (20) GD-LRS (20) GD-HES130/0.4 (20)	60	GI surgery	Goal-directed HES 130/0.4 reduced time to pass flatus (6 hours compared to restricted group and 9 hours compared to GD-LRS)

HES = hydroxyethyl starch, LRS = lactated Ringer's solution, GI = gastrointestinal, and GD = goal-directed therapy.

**Table 10:** Overview of randomized control trials related to PONV by primary outcome.

Author, year	Fluid compared	*N*	Operation	Conclusion
*Primary outcome: PONV*				
Chaudhary [19], 2008	LRS 2 mL/kg LRS 12 mL/kg 4.5% hetastarch 12 mL/kg	60	Open cholecystectomy	Preoperative fluid supplement rate (12 mL/kg) (both colloid and crystalloid) decreases incidence of PONV, vomiting, and use of antiemetic
Mishra [36], 2017	NSS 5% dextrose	100	Laparoscopic cholecystectomy	5% dextrose fluid reduced incidence of PONV, but not vomiting
Rao [42], 2017	LRS 5% dextrose	112	Laparoscopic cholecystectomy	Postoperative IV loading 1000 mL of 5% dextrose fluid reduced incidence of PONV, but not vomiting

*Primary outcome was another objective but also had these outcome*				
Ghodraty [22], 2017	6% HES130/0.4 (Voluven) LRS	91	GI surgery	HES 130/0.4 reduced incidence of vomiting, but not PONV

PONV = postoperative nausea vomiting, HES = hydroxyethyl starch, NSS = normal saline solution, LRS = lactated Ringer's solution, and GI = gastrointestinal.

## Data Availability

All data collected in this research are available for review.

## Supplementary Materials

Supplementary Materials The first one is “Full search term,” which consists of full search terms we used on an electronic database; it is cited as “Appendix S1” in the manuscript. The second one is “Data sheet,” which consists of full results from our systematic review; it is cited as “Table S1” in the manuscript.
