# Supplementary material for: Intravenous Fluid of Choice in Major Abdominal Surgery: A Systematic Review
Source: Crit Care Res Pract. 2020 Aug 3;2020:2170828. doi: 10.1155/2020/2170828 (PMC7421038; doi:10.1155/2020/2170828)
Supplement: Supplementary Materials — The first one is “Full search term,” which consists of full search terms we used on an electronic database; it is cited as “Appendix S1” in the manuscript. The second one is “Data sheet,” which consists of full results from our systematic review; it is cited as “Table S1” in the manuscript. [file 2170828.f1.pdf]

Appendix S1. Full search term

A full search term we used on an electronic database.

PUBMED : 973

("Vascular Surgical Procedures"[Mesh] OR "Cytoreduction Surgical Procedures"[Mesh] OR "Digestive System Surgical Procedures"[Mesh:NoExp] OR "Anastomosis, Roux-en-Y"[Mesh] OR "Appendectomy"[Mesh] OR "Biliary Tract Surgical Procedures"[Mesh:NoExp] OR "Cholecystectomy"[Mesh] OR "Biliopancreatic Diversion"[Mesh] OR "Colectomy"[Mesh] OR "Gastrectomy"[Mesh:NoExp] OR "Gastroenterostomy"[Mesh] OR "Hepatectomy"[Mesh] OR "Liver Transplantation"[Mesh] OR "Pancreas Transplantation"[Mesh] OR "Pancreatectomy"[Mesh] OR "Pancreaticoduodenectomy"[Mesh] OR "Pancreaticojejunostomy"[Mesh] OR "Proctectomy"[Mesh] OR "Laparotomy"[Mesh] OR "Pelvic Exenteration"[Mesh] OR "Splenectomy"[Mesh] OR "Gynecologic Surgical Procedures"[Mesh:NoExp] OR "Hysterectomy"[Mesh:NoExp] OR "Ovariectomy"[Mesh] OR "Salpingectomy"[Mesh] OR "Uterine Myomectomy"[Mesh] OR "Urologic Surgical Procedures"[Mesh:NoExp] OR "Cystectomy"[Mesh] OR "Kidney Transplantation"[Mesh] OR "Nephrectomy"[Mesh] OR "Prostatectomy"[Mesh] OR "Aortic Aneurysm, Abdominal/surgery"[Mesh] OR "Laparoscopy"[Mesh] OR Abdominal surgery*[tiab] OR Pelvic surgery*[tiab] OR Laparotomy*[tiab] OR Laparoscopy*[tiab] OR Roux-en-Y*[tiab] OR Roux en Y*[tiab] OR Appendectom*[tiab] OR Gastrectom*[tiab] OR Colectom*[tiab] OR Anterior resection*[tiab] OR Sigmoidectom*[tiab] OR Proctectom*[tiab] OR Abdominoperineal resection*[tiab] OR APR[tiab] OR AR[tiab] OR cholecystectom*[tiab] OR Hepatectom*[tiab] OR Pancreaticoduodenectom*[tiab] OR PPPD*[tiab] OR PRPD*[tiab] OR Pancreatectom*[tiab] OR liver resection*[tiab] OR liver transplant*[tiab] OR hepatic transplant*[tiab] OR Splenectom*[tiab] OR Hysterectom*[tiab] OR Ovariectom*[tiab] OR Salpingo-oophorectom*[tiab] OR salpingectomy*[tiab] OR Surgical staging*[tiab] OR Cystectom*[tiab] OR Kidney transplant*[tiab] OR Renal transplant*[tiab] OR Nephrectom*[tiab] OR nephroureterectom*[tiab] OR Prostatectom*[tiab]) AND (("Fluid Therapy/adverse effects"[Mesh] OR "Fluid Therapy/standards"[Mesh] OR "Fluid Therapy/therapy"[Mesh]) OR ("Isotonic Solutions/adverse effects"[Mesh] OR "Isotonic Solutions/standards"[Mesh] OR "Isotonic Solutions/therapeutic use"[Mesh]) OR ("Sodium Chloride/adverse effects"[Mesh] OR "Sodium Chloride/standards"[Mesh] OR "Sodium Chloride/therapeutic use"[Mesh]) OR ("Glucose/adverse effects"[Mesh] OR "Glucose/standards"[Mesh] OR "Glucose/therapeutic use"[Mesh]) OR "Ringer's acetate" [Supplementary Concept] OR ("Colloids/adverse effects"[Mesh] OR "Colloids/standards"[Mesh] OR "Colloids/therapeutic use"[Mesh]) OR ("Plasma Substitutes/adverse effects"[Mesh] OR "Plasma Substitutes/standards"[Mesh] OR "Plasma Substitutes/therapeutic use"[Mesh]) OR ("Hydroxyethyl Starch Derivatives/adverse effects"[Mesh] OR "Hydroxyethyl Starch Derivatives/standards"[Mesh] OR "Hydroxyethyl Starch Derivatives/therapeutic use"[Mesh]) OR ("Serum Albumin, Human/adverse effects"[Mesh] OR "Serum Albumin, Human/standards"[Mesh] OR "Serum Albumin, Human/therapeutic use"[Mesh]) OR "Plasmalyte R" [Supplementary Concept] OR "Plasmalyte A" [Supplementary Concept] OR ("Polygeline/adverse effects"[Mesh] OR "Polygeline/standards"[Mesh] OR "Polygeline/therapeutic use"[Mesh]) OR (

"Dextrans/adverse effects"[Mesh] OR "Dextrans/standards"[Mesh] OR "Dextrans/therapeutic use"[Mesh]) OR NSS*[tiab] OR 0.9% Saline*[tiab] OR 0.9% NaCl*[tiab] OR Normal Saline*[tiab] OR Sodium Chloride Solution*[tiab] OR Dextrose*[tiab] OR Glucose*[tiab] OR LRS[tiab] OR LAS[tiab] OR LA[tiab] OR LR[tiab] OR Lactated Ringer*[tiab] OR Ringers Lactate*[tiab] OR Ringer's, Lactate*[tiab] OR Lactate, Ringer*[tiab] OR Hartmann's Solution*[tiab] OR Ringer Solution*[tiab] OR Ringers Solution*[tiab] OR Ringer’s Solution*[tiab] OR Ringer's acetate*[tiab] OR Acetated Ringer*[tiab] OR Dextrose*[tiab] OR glucose*[tiab] OR Hydroxyethyl Starch*[tiab] OR HES*[tiab] OR HAES*[tiab] OR Voluven[tiab] OR Venofundin*[tiab] OR Hetastarch*[tiab] OR Pentaspan*[tiab] OR Pentastarch*[tiab] OR Volulyte*[tiab] OR Tetraspan*[tiab] OR Colloid*[tiab] OR Albumin*[tiab] OR Plasmalyte*[tiab] OR Dextran*[tiab] OR Gelofusin*[tiab] OR Gelatin*[tiab] OR Polygelin*[tiab] OR Haemaccel*[tiab] OR Gelaspan*[tiab] OR Plasmion*[tiab] OR Geloplasma*[tiab] OR Thomaegelin*[tiab]) AND ("Water-Electrolyte Imbalance "[Mesh] OR "Intraoperative Complications"[Mesh] OR "Postoperative Complications"[Mesh] OR "Heart Diseases "[Mesh] OR "Acute Kidney Injury "[Mesh] OR "Length of Stay"[Mesh] OR Hypercalcemia*[tiab] OR Hyperkalemia*[tiab] OR Hypernatremia*[tiab] OR Hypocalcemia*[tiab] OR Hypokalemia*[tiab] OR Hyponatremia*[tiab] OR Volume overload*[tiab] OR Hypervolum*[tiab] OR Electrolyte imbalance*[tiab] OR Abnormal electro*[tiab] OR Blood loss*[tiab] OR Bleeding*[tiab] OR haemorrhage*[tiab] OR hemorrhage*[tiab] OR Acute kidney injury*[tiab] OR AKI*[tiab] OR Acute renal failure*[tiab] OR MACE*[tiab] OR major adverse cardiac event*[tiab] OR Myocardial infarction*[tiab] OR respiratory failure*[tiab] OR Mortality*[tiab] OR Dead*[tiab] OR death*[tiab] OR Length of stay*[tiab]) NOT (CABG[*tiab] OR Coronary bypass*[*tiab])

Embase (OVID) : 439

((vascular surgical procedures/ or cytoreduction surgical procedures/ or digestive system surgical procedures/ or anastomosis, roux-en-y/ or appendectomy/ or biliary tract surgical procedures/ or cholecystectomy/ or colectomy/ or proctocolectomy, restorative/ or gastrectomy/ or gastric bypass/ or hepatectomy/ or liver transplantation/ or pancreas transplantation/ or pancreatectomy/ or pancreaticoduodenectomy/ or laparotomy/ or pelvic exenteration/ or splenectomy/ or gynecologic surgical procedures/ or hysterectomy/ or ovariectomy/ or salpingo-oophorectomy/ or salpingectomy/ or uterine myomectomy/ or urologic surgical procedures/ or cystectomy/ or kidney transplantation/ or nephrectomy/ or nephroureterectomy/ or prostatectomy/) or (Abdominal surgery* or Pelvic surgery* or Laparotomy* or Laparoscopy* or Roux-en-Y* or Roux en Y* or Appendectom* or Gastrectom* or Colectom* or Anterior resection* or Sigmoidectom* or Proctectom* or Abdominoperineal resection* or APR or AR or cholecystectom* or Hepatectom* or Pancreaticoduodenectom* or PPPD* or PRPD* or Pancreatectom* or liver resection* or liver transplant* or hepatic transplant* or Splenectom* or Hysterectom* or Ovariectom* or Salpingo-oophorectom* or salpingectomy* or Surgical staging* or Cystectom* or Kidney transplant* or Renal transplant* or Nephrectom* or nephroureterectom* or Prostatectom*).ti,ab,kw.) and ((Glucose/ae, st, tu or Sodium Chloride/ae, st, tu or Colloids/ae, st, tu or Serum Albumin, Human/ae, tu or exp Plasma Substitutes/ae, st, tu or exp Isotonic Solutions/ae, st, tu) or (NSS* or “0.9% Saline” or “0.9% NaCl” or Normal Saline* or Sodium

Chloride Solution* or Dextrose* or Glucose* or LRS or LAS or LA or LR or Lactated Ringer* or Ringers Lactate* or Ringer's, Lactate* or Lactate, Ringer* or Hartmann's Solution* or Ringer Solution* or Ringers Solution* or Ringer's Solution* or Ringer's acetate* or Acetated Ringer* or Dextrose* or glucose* or Hydroxyethyl Starch* or HES* or HAES* or Voluven or Venofundin* or Hetastarch* or Pentaspan* or Pentastarch* or Volulyte* or Tetraspan* or Colloid* or Albumin* or Plasmalyte* or Dextran* or Gelofusin* or Gelatin* or Polygelin* or Haemaccel* or Gelaspan* or Plasmion* or Geloplasma* or Thomaegelin*).ti,ab,kw.) and ((water-electrolyte imbalance/ or hypercalcemia/ or hyperkalemia/ or hypernatremia/ or hypocalcemia/ or hypokalemia/ or hyponatremia/ or water intoxication/ or intraoperative complications/ or blood loss, surgical/ or postoperative complications/ or postoperative hemorrhage/ or shock, surgical/ or heart diseases/ or heart arrest/ or heart failure/ or myocardial ischemia/ or acute coronary syndrome/ or angina pectoris/ or myocardial infarction/ or renal insufficiency/ or acute kidney injury/ or "Length of Stay"/) or (Hypercalcemia* or Hyperkalemia* or Hypernatremia* or Hypocalcemia* or Hypokalemia* or Hyponatremia* or Volume overload* or Hypervolum* or Electrolyte imbalance* or Abnormal electro* or Blood loss* or Bleeding* or haemorrhage* or hemorrhage* Acute kidney injury* or AKI* or Acute renal failure* or MACE* or major adverse cardiac event* or Myocardial infarction* or respiratory failure* or Mortality* or Dead* or death* or Length of stay*).ti,ab,kw.)

Table S1 Data sheet, Full result from our systematic review

Author (year)	Study design	Fluid compared, role (n)	Surgery	Study conclusion	Additional outcomes
Potura, E etal, 2015[40], Austria	RCT, open lebel	-NSS (C) n=76<br>-Acetate-buffered crystalloid [Elomel-Isoton](I) n=72<br>-Intra-postoperative maintenance	Cadaveric renal transplantation	A chloride-reduced, acetate-buffered crystalloid during cadaveric renal transplantation result in less hyperchloremia and consequent hyperchloremic metabolic acidosis compared to NSS	-Hyperkalemia >5.9(%): 17 vs 21 ; p = 0.56<br>-Minimum base access (mmol/L): -4.5 (-6 to -2.4) vs -2.6 (-4 to -1) ; p<0.001<br>-Maximum chloride (mmol/L): 109 (107-111) vs 107 (105-109); p<0.001<br>-Need cathecolamine (%) 30 vs 15 ; p = 0.0278<br>-AKI : no different in BUN,Cr, urine output in POD 1, 3 and7
Zhu, Q.L.,2018[57] Chinese	RCT	-LRS n=25<br>-Succinylated gelatin group n =21<br>-HES 40 n=24<br>-Preoperative volume expander	-Laparoscopic colorectal surgery<br>-Elder 65-85 yrs<br>-ASA I, II	Compare with LRS and hypertonic NaCl HES, succinylated gelatin solution can maintain good splanchnic perfusion, even after a long period of pneumoperitoneum (60 min).	-Gastric pH : in gelatin group always >7.32, but other 2 lower at T5-T8<br>-PgaCO2 of group R in T4-T7 and group H in T5 drop from baseline<br>-Blood loss(mL): 81+/-49 vs 103+/-38 vs 93+/-80; p>0.05<br>-CVP : group R loss CVP at T6<br>-K not different<br>-LOHs, MACE not report
Rasmussen, K. C.[44], 2015, Denmark	RCT	-6%Dextran 70 n=19<br>-LRS n=18<br>-Intraoperative volume expander	Cystectomy	-Coagulation competence was evaluated by TEG and the perioperative blood loss was related to reduction in MA by 25 %.	-TEG MA (mm): 48.9 (44.6-53.2) vs 62.1 (58.9-65.3); p=0.001<br>-Blood loss(ml): 2339+/-1470 vs 1822+/-1240; p=0.27<br>-Bleed > 1500 ml(n): 11 vs 4, p=0.04<br>-Fluid balance(ml): 1520+/-668 vs 1925+/-696; p=0.03<br>-LOHs (days): 9(5-24) vs 7 (6-92); p=0.69
Modi, M. P.[37],2012 ,Saudi arabia	RCT	-LRS n=37<br>-NSS n=37	Living donor kidney transplantation.	-LRS is safe and may be superior to NSS in live renal transplant because it	-HCO3 (mmol/L): 21.62+/-3.56 vs 19.47+/-3.02; p<0.05<br>-K (mmol/L): 3.99+/-0.71 vs 4.31+/-0.59; p<0.05<br>-Cl (mmol/L): 98.5+/-3.03 vs 103.92+/-4.25; p<0.05

		-Intraoperative maintenance		avoids metabolic acidosis and hyperkalemia	-no different in AKI
Kammerer, T.[26], 2018, Germany	RCT	-5% Albumin n=53<br>-6% HES 130/0.4 [Volulyte] n=47<br>-Intra-post operative Volume expander	Cystectomy with ileum conduit or neobladder	-Perioperative 5% albumin and balanced 6% HES solutions have comparable safety profiles with respect to renal function in these patients.	-AKI (Cystatin C ratio at day 90) : 1.11 (1.01-1.23) vs 1.08 (1.00-1.20); p=0.165<br>-GFR decrease (%): 11.46 vs 8.96; p=0.79<br>-EBL (ml): 1126+/-549 vs 1127+/-618; p=0.771
Kim, S. Y.[29],2013, Korea	RCT	-Plasmalyte n=30<br>-NSS n=30<br>-Intraoperative maintenance	Living donor kidney transplantation.	NS and Plasmalyte can be used safely in patients undergoing uncomplicated living donor kidney transplantation. However, NS should be used with caution in kidney transplant patients who present preoperatively with metabolic acidosis.	-Base excess (T3): -2.3+/-3.3 vs -6+/-4.2; p<0.05<br>-AKI (Cr, Cl, urine output): not different in POD7<br>-Graft failure: 3 VS 1; p=0.301<br>-LOHs (days): 16.2 vs 16.8
Ghodraty, M. R.[22], 2017,	RCT, single blind	-LRS n=45<br>-6%HES130/0.4 (voluven) n=46<br>-Intraoperative volume expander	Gastrointestinal operation for resection and anastomosis<br>ASA I II III	Colloid fluids may have a preventive role in gastrointestinal operations regarding reduction of postoperative ileus	-Ileus time (h): 86.7+/-23.6 vs 73.4+/-20.8; p=0.006<br>-PONV (%): 37.8 vs 26.1;p=0.266<br>-Vomiting (%): 11 vs 3; p=0.022<br>-AKI: no different between group
Kancir, A. S.[27], 2015, Denmark	RCT, double blinded	-6% HES130/0.4 (Voluven) n=18<br>-NSS n=18<br>-Intraoperative volume expander	Radical prostatectomy	HES doesn't lead to nephrotoxicity in patients undergoing prostatectomy with normal preoperative renal function and stable hemodynamic. Also HES has a greater volume expansion effect; there is increased blood loss in the HES group	-U-NGAL at DC (ng/μmol): 4(4-6) vs 5(3-7); p=0.474<br>-EBL (mL): 1256(669) vs 747(331); p=0.008<br>-Other parameter show good volume expansion in HES<br>-LOHS (days): 2.5 (2.5-2.8) vs 2.5 (2.5-2.5); P=0.338
Rao, V.[42],2017, india	RCT	-5%Dextrose n=56<br>-LRS n=59<br>-Postoperative expander	Laparoscopic cholecystectomy	Postoperative administration of 5% dextrose solution after emergence from general anesthesia reduces the incidence of PONV and improve well-being at postop 24 hours	-PONV within 24hrs (n,%): 24(43%) vs 43(73%); p<0.001<br>-Vomiting within 24hrs (n,%): 8(14%) vs 10(17%); p<0.698<br>-Improve hunger and fatigue in 6 hours<br>-DTx (mg%,SD): 282(32) vs 92(8); p<0.001

Jin, S. L.[25],2010, China	RCT	-LRS n=12<br>-6%HES130/0.4 (Voluven) n=12<br>-4%Modified GEL (Gelofusine) n=12<br>-Preoperative volume expander	Gastrectomy<br>ASA I-II	Gelatin reduced clot quality associated with derangements of fibrin polymerization and HES 130/0.4 delayed initiation of sufficient thrombin generation to convert fibrinogen to fibrin and impaired platelet function.	-TEG R after AHFI(min): 11.4+/-2.1 vs 20.7+/-3.8 vs 14.5+/-2.5;p(HES) <0.05<br>-TEG K after AHFI (min): 5.3+/-1.1 vs 8.3+/-2.4 vs 6.8+/-2.5;p(HES) <0.05<br>-TEG angle after AHFI : 40.9+/-6.9 vs 27.1+/-6.3 vs 34.3+/-5.6; p(HES) <0.05<br>-TEG MA postop (mm): 59.4+/-5.6 vs 52.9+/-4.8 vs 49.5+/-6.7; p(GEL)<0.05<br>-vWF after AHFI(%) : 143.8+/-28.8 vs 110.2+/-23.6 133.3+/-38.6; p(HES) <0.05<br>-VIII:C (%) after AHFI : 136.4+/-28.5 vs 99.3+/-20.9 vs 110.3+/-23.4; p(HES) <0.05<br>-Blood loss: 321+/-84 vs 349+/-98 vs 314+/-58 ; p=NS<br>-PT,PTT,Fibrinogen: not different<br>-not report Morbid/mortality, AKI
Rittoo, D.[47],2005, UK	RCT	-6%HES 200/0.6 (eloHAEs) n =20<br>-4%Gelofusine n=20<br>-Intraoperative volume expander	Elective open infrarenal AAA repair	HES may damp down the systemic inflammatory response and reduce endothelial cell dysfunction but may increase risk of bleeding due to lower vWF level	-Colloid volume (ml): 3000(2500-3437) vs 3500(3063-4213); p<0.01<br>-also less crystalloid need<br>-Platelet count at POD1 (x10^3): 121(101-141) vs 147(128-170); p<0.05<br>-CRP (mg/L): 142(113-196) vs 246(189-291); p<0.01<br>-Ualb when off clamp (mg/mmol) :9.3 vs 23.3; p<0.05<br>-vWF at 24 hrs(U/dL): 81 vs 209; p< 0.001
Deng, Y.[21],2017, Chinese	RCT	-LRS n=12<br>-Succinylated gelatin n=12<br>-4.2% NaCl in 7.6% hydroxyethyl starch 40 n=12	Laparoscopic colorectal surgery<br>ASA I,II<br>AHFI	Hypervolemic infusion of these solutions during the induction of anesthesia can improve gastric mucosal perfusion. HS can maintain a more stable hemodynamic effect when used with	-No different in Pg-aCO2 among 3 group of patient<br>-HS-HES can prolong effect of volume expansion and decrease SVRI<br>-HS-HES can increase Na which gradually decrease at post op<br>-pH, BE, CO2 not different

		Preop volume expander		caution in patients with preoperative hypernatremia.	
Weinberg, L.[54], 2017, Australia	RCT double blind	-NSS n=25<br>-Plasma-lyte n=24<br>-Intra-postoperative maintenance	Cadaveric renal transplantation	Use of NS intra-postoperative for 48 hours was associated with hyperchloraemic acidosis and hyperkalaemia, with greater use of medical interventions compared to PL. These biochemical differences were not associated with adverse clinical outcomes	-Hyperkalemia at 48 h (%): 80 vs 50; risk different 0.3(0.05-0.55) p=0.04<br>-Hemodialysis for hyperK (n) : 13 vs 4; p=0.02<br>-LOHs (day): 6.5 (6, 13.2) vs7.1 (6, 10.4); p=0.18<br>-CRR: -0.03(-0.17,0.1) vs 0.18(0.03,0.34); p=0.04<br>-Higher Cl, and lower HCO3 in NS group<br>-no different in DGF and need for hemodialysis at 7 days
Khajavi, M. R.[28], 2008, Iraq	RCT, double blind	-NSS n=26<br>-LRS n=26<br>-Intraoperative fluid maintenance	Living donor kidney transplantation.	Compared with NS, LR infusion may lead to a lower serum potassium level and a lower risk of acidosis, while there is major concern of the hypercoagulable state in these patients.	-K at the end of operation (meq/L): 4.8+/-0.7 vs 4+/-0.8; p<0.001<br>-K change (meq/L): +0.5+/-0.6 vs -0.5+/-0.9; p<0.001<br>-pH at the end of operation (unit): 7.29+/-0.08 vs 7.34+/-0.05; p=0.007<br>-pH change (unit): -0.06+/-0.05 vs -0.005+/-0.007; p=0.001<br>-Vascular thrombosis (n,%): 0 vs 2(7.7); p=0.49
Weinberg, L.[53], 2015, Australia	RCT, multicenter, double blind	-Plasma-lyte n=30<br>-Harttman's solution n=30<br>-Intraoperative maintenance	Open major hepatic resection<br>ASA I,II,III	In liver resection patients, HS and PL led to similar base excess values but different postoperative plasma biochemistry and hematology values.	-SBE at postoperative: -0.9(2.3) vs -1.7(2.2); p=0.17<br>-Lactate at postoperative: 1.9(1.13) vs 2.9(1.76); p=0.02<br>-Chloride at postoperative: 106(2.4) vs -108(3); p=0.01<br>-Magnesium at postoperative: -0.85(0.12) vs 0.71(0.11); p<0.001<br>-MACE: myocardial infarction, n=0 vs n=4; - arrhythmia, n=2 vs n=3<br>-AKI: no difference in renal complications<br>-Blood loss (ml): 300 (200–413) vs 500 ml (300–638); P=0.03<br>-LOHS (days): 5.9 vs 7.8; p=0.04<br>-Thirty-day mortality: n=0 vs n=2; p=0.9

Liang, H.[31],2010, China	RCT	-6%HES 200/0.5 (HAES-steril6%) n=17<br>-6%HES 130/0.4 (Voluven) n=18<br>-Preloading infusion	Laparoscopic-assisted radical colectomy	Preloading infusion with HES 200/0.5 can inhibit excessive platelet activation, and has a more significant effect of alleviating hypercoagulable state than HES 130/0.4 during perioperative period.	-TEG R (min): 4.8+/-1.5 vs 4.3+/-1.3; p<0.05<br>-TEG MA (mm): 67+/-6 vs 74+/-6; p<0.05<br>-GPIIb/IIIa and CD62P was expressed lower in HES200 group<br>-no significant in blood loss or other complication
Sander, O.[48],2003, Germany	RCT, double blind	-6%HES 200/0.5 n=27<br>-6%HES 130/0.4 n=29<br>-Intra-postoperative volume expander	Major gynecologic surgery	Therapeutic equivalence of this novel low-substituted HES 130/0.4 solution and a standard HES 200/0.5 solution for perioperative volume replacement.	-Colloid volume (ml): 1189+/-610 vs 1224+/-544 [equivalence bound 889-1889]<br>-Hct at post operation 6 hrs (%): 0.3+/-0.05 vs 0.33+/-0.04 p< 0.05<br>-INR at post operation 6 hrs: 1.25+/-0.19 vs 1.18+/-0.09; p<0.05<br>-LOHS (day,IQR): 14(9) vs 16(12)
Senagore, A. J.[49], 2009, USA	RCT, double blind, 3 arm study	-STD n=22<br>-GD LR n=21<br>-GD H: 6%Hetastatch in balanced salt n=21<br>-Intraoperative expander	Laparoscopic segmental colectomy	Goal-directed fluid management with a colloid/balanced salt solution offers no advantage and is more costly	-Volume to achieve stroke volume (863+/-850 ml vs 389+/-287 ml; p < 0.05)<br>-Mean complication per patient: 1.82 vs 1.95 vs 4.38<br>-LOHS (hours): 64.9 vs 71.8 vs 75.5; p<0.05<br>-Mean number of complication: 1.82+/-1.2 vs 2+/-1.7 vs 4.4+/-4<br>-Arrythmia: 2 vs 1 vs 3<br>-Mortality: 0 vs 0 vs 1
Marik, P. E.[35], 1997, USA	RCT, open lebel	-LRS n=15<br>-Hetastarch (Hespan) n=15<br>-Intra-postoperative volume expander	Elective open AAA repair	Patients undergoing major surgery, volume resuscitation with HES may improve microvascular blood flow and tissue oxigination	-Maximum pH decrease: 0.13+/-0.04 vs 0.07+/-0.03; p <0.001<br>-Blood loss (mL): 1509+/-939 vs 1503+/-890; p=1<br>-Maximum change PTT from baseline (sec): 2.3+/-3 vs 5.6+/-5.5; p=0.1<br>-LOHs (day): 6.4+/-1.7 vs 5.6+/-1.3; p=0.3
Ragaller, M.[41], 2000, Germany	RCT, double blind	-6%HES200/0.5 in 7.2% NaCl n=16<br>-6%HES200/0.5 in 0.9% NaCl n=13	Elective open AAA repair	Hypertonic HES applied in a titrated fashion restored hemodynamic stability faster and without volume overload	-Colloid use for restore best PCWP (ml, mL/kg): 162+/-111vs 265+/-108; p=0.013<br>-Time to restore best PCWP (min): 9 vs 14; p=0.073

		Intraoperative volume expander			-no different in blood chemistry and intraoperative hemodynamic
Rasmussen, K. C.[43], 2014, Denmark	RCT, double blind	-6%HES130/0.4 (Voluven) n=17<br>-LRS n=16<br>-Intraoperative volume expander	Cystectomy	HES 130/0.4 reduced clot strength and perioperative hemorrhage increased by more than 50%, while administration of lactated Ringer's solution provoked an approximately 2.5 times greater positive volume balance at the end of surgery.	-TEG: significant higher in HES group (MA, degree)<br>-Platelet (x10^9/L): 154(31) vs 217(49);p=0.02<br>-Fibrinogen (mmol/L): 5.09 [1.79] vs 7.68 [2.11]; p=0.001<br>-Blood loss (ml): 2181 ± 1190 vs 1370 ± 603; P=0.038<br>-Positive fluid (ml): 995(35-2180) vs 2478(1230-3880); p=0.0001<br>-AKI: no differences in renal complications<br>-LOHs (days,SD): 9.6(3.3) vs 12.1(10.3)
Lavu, H.[30], 2014, USA	RCT, double blind	-LRS n=128<br>-3%Hypertonic saline n=131<br>-Intra-postoperative maintenance	Pancreaticoduodenectomy	A moderately restrictive fluid regimen with HYS resulted in a statistically significant 25% reduction in complications when adjusted for age, weight, and pancreatic texture.	-Complication(%): 54 vs 43 (RR=0.75, 0.62-1.02); p=0.073<br>-Adjust with pancreatic gland texture: 21% reduction in HYS<br>-Adjust with pancreatic gland, age, weight: 25% reduction in HYS<br>-total IV input (mL/kg): 315 vs 278 ml; p=0.017<br>-EBL (mL): 400(50-6900) vs 350(45-2250); p=0.99<br>-MACE and AKI not different<br>-LOHS (days): 7(4-41) vs 7(5-61)<br>-Mortality 90 days (%) : 4 vs 3
Demir, A.[20], 2015, Turkey	RCT	-6%HES 130/0.4 (18)<br>-4% gelatin [gelofusine] (18)<br>-Intraoperative volume expander	Living donor liver transplantation.	Gelofusine seem to cause more impairment in renal functions in elective living-donor liver transplantation.	-Preoperative and postoperative GFR significant decrease in gelofusine group; p<0.001<br>-AKI :2 vs 5<br>-Mortality 90 days (n) : 1 vs 2; p=1<br>-LOHS (days): 17.8+/-7.09 vs 19.1+/-10.4; p=0.92
Loffel, L. M.[32], 2016, Switzerland	RCT, single center, double blind , parallel	-Chloride-depleted glucose solution 5% (G5K) n=22	Open radical cystectomy with urinary diversion	Perioperative administration of G5K did not enhance first defecation, but may accelerate recovery of normal	-1st defecation(h): 93(19 to 168) vs 120 (43 to 241), group difference, -16 (95% CI, -38 to 6); p= 0.173<br>-Return to bowel function: 138(54 to 262 vs 169(108 to 318); group difference, -38 (95% CI, -74 to -12);

		-Ringer's maleate solution n=22<br>-Intra-postoperative maintenance		gastrointestinal function, and reduces potassium and magnesium substitution	p=0.004<br>-MACE: 5 (22.7) vs 2 (9.1)<br>-AKI: no difference in renal complications<br>-Blood loss (ml): 880 (200–1800) vs 1200 (200–2200); p=0.135<br>-LOS (days): 14.5 (10–23) vs 15.5 (11–26); p=0.233
Rasmussen, K. C.[45], 2016, Denmark	RCT, double blind	-5%Human albumin n=19<br>-LRS n=20	Open radical cystectomy with urinary diversion	5% HA compared to LR reduces the postoperative volume surplus but affects coagulation competence, but not to an extent that albumin affects bleeding or outcome measures	-TEG MA (mm): 60(7) vs 68 (6); p<0.002<br>-Bleeding associate with lower TEG-MA<br>-Blood loss (mL): 1658(800-3300) vs 1472 (700-4330); p=0.45<br>-Fluid balance (mL): 1095(400-2460) vs 1687(415-2850); p<0.001<br>-AKI : no different<br>-LOHS (days): 8 (5–90) vs 7 (3–20) days; P=0.27
Szturz, P.[50], 2014, Czech	RCT	-LRS n=57<br>-6%HES130/0.4 n=58<br>-Intraoperative volume expander	Major urological surgery	crystalloids and colloids are effective in correcting flow-related perfusion abnormalities, but different characteristics such as unequal distribution between compartments.	-Total intraoperative intake(mL): 5000 vs 2200; p=0.001<br>-Gastrointestinal dysfunction(%): 31.6 vs 15.5; p=0.05<br>-AKI: no different<br>-LOHS : not different
Zhang, J.[56], 2012, China	RCT, double blind	-R-LRS n=20<br>-GD-RL n=20<br>-GD-C (6%HES 130/0.4) n=20<br>-Intraoperative volume expander	Gastrointestinal surgery	intraoperative treatment with PPV-directed 6% HES (130/0.4) leads to more stable hemodynamics, faster bowel function recovery and a shorter postoperative hospital stay than treatment with restrictive or PPV-directed lactated Ringer's solution	-LOHS (days): 10.9+/-1.2 vs 11.9+/-1.2 vs 10.9+/-1.2; p<0.001<br>-Time to flatus (h): 92.1+/-9.7 vs 95.4+/-9.1 vs 86.2+/-7.2; p<0.03<br>-Infusion volume (mL): 1265 vs 2109 vs 1742; p<0.07<br>-Blood loss (mL): 256+/-139.3 vs 265+/-46.2 vs 252.5+/-44.4; p=NS<br>-Crystalloid : Colloid ratio = 1.67 :1<br>-AKI : not different between group<br>-MACE : not different
Ando, Y.[18], 2008, Japan	RCT	-6% HES 70/0.5 (Hespander) n=10<br>-Acetate ringer n=10	Abdominal surgery<br>ASA I	HES does not improve microvascular hyperpermeability, the expansion of	-Total IV (mL): 3953(3208,5093) vs 4152(3533,4599)<br>-MACR, GFR : not different between group through study period

		-Intra-postoperative maintenance		the intravascular volume by HES results in higher urinary<br>output in the postoperative period than that seen with crystalloid<br>solution	-sICAM-1 : significant lower in HES group during immediate postoperation<br>-MACE: arrhythmia: n=1 vs n=1<br>-Blood loss (ml): 115 (90–200) vs 110 (93–193)<br>-ICU LOS (h): 20 (18–20) vs 22 (20–23); P=0.02<br>-LOHS (days): 124 (21–25) vs 31 (23–37)
Yuan, X. Y.[55], 2008, China	RCT, open lebel	-20% human albumin n=64<br>-NSS n=63<br>-Post operative supplement	Abdominal surgery	albumin administration in the early stage of postoperative hypoalbuminemia is not beneficial in correcting hypoalbuminemia or for improving clinical outcomes.	-albumin level (g/dL) at POD7 : 3.83+/-0.47 vs 3.86+/-0.37; p>0.654<br>-albumin and other nutrition maker level are same along POD 0-7<br>-Immediate postop serum albumin level decrease significant compare to preop<br>-MACE : 0vs0<br>-AKI : not different<br>-Complication: 15 vs 8; p=0.116
Mishra, A.[36], 2017, India	RCT, double blind	-NSS n=50<br>-5%Dextrose n=50<br>-Intra-postoperative maintenance	Laparoscopic cholecystectomy<br>ASA I-II	Perioperative administration of 5% dextrose in patients undergoing laparoscopic surgery can reduce PONV significantly	-PONV (n,%): 33(66) vs 14(28); p<0.001<br>-Need high dose antiemetic (n,%): 9(18) vs 1(2)
Hung, M. H.[23], 2014, Taiwan	RCT, double blind	-6%HES 130/0.4 (Voluven) n=41<br>-LRS n=39<br>-Intraoperative maintenance	Major abdominal surgery<br>ASA I, II	HES 130/0.4 is a more efficient intravascular volume expander to maintain tissue perfusion than crystalloid. Transient hypocoagulability induced by HES 130/0.4 does not cause blood loss	-Blood loss (mL): 208.5+/-150.6 vs 421+/-597.1; p=0.015<br>-Total IVF (mL) 1547.9+/-424 vs 2303.1+/-1033.7; p<0001<br>-PRC transfuse (u); 2 vs 24; p=0.037<br>-TEG-K, angle, MA, G : significant decrease after infuse IV 15 mL/kg in HES group, but return to normal at 24 h
Waters, J. H.[52], 2001, USA	RCT	-NSS n=33<br>-LRS n=33<br>-Intraoperative maintenance and volume expander	Elective open AAA repair<br>Average ASA III	The use of saline was associated with more acidosis compared to the Ringer's lactate group, but with no differences in clinical outcome	-Different of pre and postoperative<br>pH: -0.08 vs -0.02; p<0.05<br>BE: -2.4 vs -4.2;p <0.05<br>HCO3: -2.2 vs -2.9; p<0.05<br>Cl: 2 vs 9; p<0.05

					-NSS use blood component more than LRS<br>-Multivariate show type off fluid not associate with complication<br>-MACE: cardiac arrhythmia, n=3 vs n=3<br>-AKI: n=4 vs n=5<br>-Blood loss (ml): 705 (335–1649) vs 1095 (430–1745)<br>-Mortality: n=1 vs n=1
Vogt, N.[51],1999, Germany	RCT	-6%HES 200/0.5 n=24<br>-5%HA n=24<br>-Intraoperative volume expander	Major urological operation<br>ASA I, II, III	6% HES 200/0.5 is an economical alternative to human colloids even in cases of large blood turnover	-No different in hemodynamic parameter<br>-TSP at end of surgery (g/L): 37.8(5.8) vs 56.9(4.7); p<0.05<br>-PTT (s): 42.4(8.8) vs 37.3(5.3); p<0.05<br>-COP(mmHg); 21.1(2.1) vs 22.5(2.2); p<0.05<br>-Blood loss(ml): 3810(1633) vs 3455(1734); p= NS<br>-Cost(euro): 482(281) vs 750(508); p<0.05<br>-AKI : not different<br>-LOHs, mortality not report
Ickx, B. E.[24], 2003, Belgium	RCT, double blind	-6%HES 130/0.4 n=20<br>-6%HES 200/0.5 n=20<br>-Intraoperative volume expansion	Major abdominal surgery<br>ASA II, III	Good immediate and medium-term plasma volume substitution effect of HES 130 compared with HES 200.	-COP postop (mmHg): 22.1(1.5) vs 21.6(1.9)<br>-Blood loss(mL): 2000(600-2800) vs 2200(500-18000)<br>-Allogeneic PRC patient(n): 5 vs 7<br>-MACE (MI) : 0 vs 1<br>-AKI : not different<br>-LOHs (days) : 15.5 (4.8) vs 14.8(3.9)
Chaudhary, S.[19], 2008, India	RCT	-LRS 2 ml/kg n=20<br>-LRS 12 ml/kg n=20<br>-4.5% HES (Hetastatch) 12 ml/kg n=20<br>-Preoperative volume expander	Elective open cholecystectomy<br>ASA I, II	Pre-operative fluid supplementation (Crystalloids and colloids 12 ml/kg) decreases the incidence of PONV equally.	-VAS for NV at 4 h: 4.3+/-3.29 vs 2.4+/-2.37 vs 2.00+/-2.53; p< 0.05 (A vs B+C)<br>-Vomiting + Anti emetic rescue (n): 18 vs 10 vs 11; p<0.05 (A vs B+C)<br>-LOHs, AKI, MACE : not report

Mahmood, A.[33], 2007, UK	RCT, double blind	-6% HES 200/0.62 (Elohes) n=21<br>-6% HES 130/0.4 (Voluven) n=21<br>-4% Gelatin (Gelofusine) n=20<br>-Intra-postoperative volume expander	Elective open infrarenal AAA repair	Compared with gelatine, volume expansion with both types of HES during AAA surgery improved renal function and reduced renal injury.	-U-IgG /cr and microalbumin/Cr significant lower in HES group in different time compare to gelatin<br>-Blood loss (mL): 1500(1055-2050) vs 1650(1025-2630) vs 1700(800-3150);p=0.760<br>-MACE : 14 vs 12 vs 12<br>-Reoperation in 24 h: 4 vs 1 vs 2<br>-RRT : 1 vs 1 vs 3<br>-30 day mortalitiy: 1 vs 1 vs 6
O'Malley, C. M.[39], 2005, USA	RCT, double blind	-NSS n=26<br>-LRS n=25<br>-Intraoperative maintenance fluid	Kidney transplant (mostly living donor)	NS did not adversely affect renal function. LR was associated with less hyperkalemia and acidosis compared with NS. LR may be a safe choice for IV fluid in kidney transplantation.	-Cr at POD3(mg/dL): 2.3+/-1.8 vs 2.1+/-1.7; p=ns<br>-Cr at PO7(mg/dL): 1.9+/-1.2 vs 1.6+/-1.3; p=ns<br>-24 h CrCl: 81+/-40 vs 94+/-30;p=ns<br>-Lowest intraoperative pH: 7.26+/-0.08 vs 7.33+/-0.07; p=0.001<br>-Lowest intraoperative HCO3: 18+/-3 vs 21+/-4; p=0.007<br>-Peak intraoperative K: 5.1+/-1.1 vs 5.1+/-0.6; p=ns<br>-End of surgery Cl : 111+/-4 vs 106+/-4; p<0.0001<br>-dialysis(n,%): 2(8) vs 1(4)<br>-LOHs : not report
Mukhtar, A.[38], 2009, Egypt	RCT	-5%Human albumin n=20<br>-6%HES130/0.4 (Voluven) n=20<br>-Intra-postoperative volume expander	Living donor liver transplant	HES 130/0.4 as an alternative to human albumin resulted in equivalent renal outcome after liver transplantation.	-Cystatin C, 24hCrCl, Cr : trend to higher in HES group but not significant<br>-Postoperative colloid use (mL): 4636+/-1153 vs 6229+/-1140; p<0.05<br>-no different in intra-postop hemodynamic<br>-Bleeding : not report<br>-Reoperation: 3 vs 1<br>-LOHs (d): 23+/-4 vs 27+/-3<br>-MACE : not report<br>-RRT: 1 vs 1<br>-Mortality: 1 vs 1

Mahmood, A.[34], 2009, UK	RCT	-6% HES 200/0.62 (Elohes) n=21<br>6% HES 130/0.4 (Voluven) n=21<br>4% Gelatin (Gelofusine) n=20<br>-Intra-postoperative volume expander	Elective open infrarenal AAA repair	HES200/0.62 provides the best splanchnic microcirculation protection, also reducing inflammation and duration of ventilation	-gastric pH at clamp removal: 7.32 vs 7.28 and 7.28; p=0.017 and 0.009<br>-Endotoxin HES vs gelatin at 12 h(%): 51 vs 37; p=0.003<br>-CRP at 48 h(mg/mL): 178 vs 223 vs 221; p<0.005<br>-Lung injury score not different<br>-Ventilator time (h): 4 vs 8 vs 11 ; p=0.012 for elohes vs gelatin<br>-Blood loss (mL): 1500(1055-2050) vs 1650(1025-2630) vs 1700(800-3150);p=0.760<br>-MACE : 14 vs 12 vs 12<br>-Reoperation in 24 h: 4 vs 1 vs 2<br>-RRT : 1 vs 1 vs 3<br>-30 day mortalitiy: 1 vs 1 vs 6
Rittoo, D.[46], 2002, UK	RCT	-6% HES 200/0.62 (Elohes) n=12<br>-4% Gelatin (Gelofusine) n=10<br>-Intraoperative volume expander	Elective open AAA repair	In AAA repair patient, plasma expansion with eloHAES improves microvascular perfusion and splanchnic oxygenation	-pH (off clamp): 7.33 vs 7.29; p=0.003<br>-Total Colloid (mL): 3175+/-607 vs 4065+/-851; p=0.01<br>-IL-6: Elohes lower than gelatin but not significant<br>-CRP at POD2: 138 vs 230; p=0.05
Joosten, A.[17], 2018, USA	RCT, double blind, multicenter	-Plasmalyte n=80<br>-Balanced colloid (Volulyte) n=80<br>-Intraoperative volume expander	Major abdominal surgery ASA I, II, III	A colloid-based goal-directed fluid therapy was associated with fewer postoperative complications than a crystalloid one.	-POMs POD2: 3(1-4) vs 2(1-3);p<0.01<br>-Major complication(%): 23 vs 9; p=0.015<br>-Anastomosis leakage: 8 vs 0; p=0.046<br>-Total intake(ml/kg/h): 9.5(6.8-11.1) vs 7.1(5.7-8.5); p<0.001<br>-Need rescue fluid(%): 24 vs 11; p=0.035<br>-Need for vasopressor (%): 89 vs 55; p<0.001<br>-SVV: 10(8-130) vs 8(7-9); p<0.001<br>-MAP: 75(72-81) vs 79(74-81); p=0.036<br>-MACE: 0 vs 2

					-RRT: 1 vs 1<br>-Mortality: 4 vs 0; p=0.25<br>-LOHs(day): 10(6-16) vs 10(6-13); p=0.43
Yates, D. R.[16], 2014, UK	RCT, double blind	-Balanced colloid (6%HES130/0.4 Volulyte) n=104<br>-Balanced crystalloid (Hartmann's solution) n=98<br>-Intraoperative volume expander	-Elective colorectal surgery<br>ASA I II III	Goal-directed fluid therapy is possible with either crystalloid or HES. There is no evidence of a benefit in using HES over crystalloid, despite its use resulting in a lower 24 h fluid balance	-Tolerate full enteral diet at POD5(%): 30 vs 32 [OR=0.96(0.52-1.77)]<br>-Time to 1st bowel movement(days): 4 vs 3.7; p=0.62<br>-IVF in first 24h(mL): 5398(4498-6401) vs 6375(5198-7535); p<0.01<br>-Recue colloid(%): 12 vs 38; p<0.001<br>-No different in inflammation parameter<br>-Blood loss (mL): 250(50-700) vs 200(100-620); p=ns<br>-TEG MA and clot formation time non-significant increase in HES<br>-Total complication(%): 46 vs 38 (OR 140(0.79-2.48))<br>-Crystalloid : colloid ration: 1.6:1<br>-MACE: 15 vs 6<br>-AKI :4 vs 0<br>-LOHs (days): 9 vs 8; p=0.74<br>-Mortality (n): 5 vs 2 [OR=1.99(0.37-10.78)]
Feldheiser, A.[15], 2013, Germany	RCT, double blind	- Balanced crystalloid (Jonosteril) n=24<br>-Balanced colloid (6%HES130/0.4 Volulyte) n=24<br>-Intraoperative volume expander	Cyroreductive surgery	Balanced HES solution is associated with better hemodynamic stability and reduced FFP need; no signs of renal impairment by colloid solutions when fluid administration is targeted to optimize cardiac preload	-Time to reach max fluid (h.m): 2.26 (1.39; 2.45) vs 3.33(2.25; 4.26);p=0.006<br>-Get maximum dose of study fluid(n,%): 22(92) vs 15(62); p=0.036<br>-AKI: none<br>-Blood loss (ml): 820 (500–2200) vs 1500 (700–2650); P=0.313<br>-LOHS (days): 13 (11–17) vs 13 (10–15); P= 0.401<br>-In-hospital mortality: n=0 vs n=1; P= 1.000<br>-Three-month mortality: n=0 vs n=5; P=0.051
